# Supplementary material for: Data reuse in agricultural genomics research: challenges and recommendations
Source: Gigascience. 2025 Jan 13;14:giae106. doi: 10.1093/gigascience/giae106 (PMC11727710; doi:10.1093/gigascience/giae106)

GigaScience

Data reuse in agricultural genomics research: challenges and recommendations

--Manuscript Draft--

**Manuscript Number:**	GIGA-D-24-00228R1
**Full Title:**	Data reuse in agricultural genomics research: challenges and recommendations
**Article Type:**	Review
**Funding Information:**	National Institute of Food and Agriculture (2020-70412-32615) — Not applicable<br>National Institute of Food and Agriculture (2021-70412-35233) — Not applicable<br>National Science Foundation (RCN #2126334) — Not applicable
**Abstract:**	The scientific community has long benefited from the opportunities provided by data reuse. Recognizing the need to identify the challenges and bottlenecks to reuse in the agricultural research community and propose solutions for them, the data reuse working group was started within the AgBioData consortium framework. Here, we identify the limitations of data standards, metadata deficiencies, data interoperability, data ownership, data availability, user skill level, resource availability, and equity issues, with a specific focus on agricultural genomics research. We propose possible solutions stakeholders could implement to mitigate and overcome these challenges and provide an optimistic perspective on the future of genomics and transcriptomics data reuse.
**Corresponding Author:**	James Koltes<br>Iowa State University of Science and Technology: Iowa State University<br>Ames, Iowa UNITED STATES
**Corresponding Author Secondary Information:**	
**Corresponding Author's Institution:**	Iowa State University of Science and Technology: Iowa State University
**Corresponding Author's Secondary Institution:**	
**First Author:**	Alenka Hafner
**First Author Secondary Information:**	
**Order of Authors:**	Alenka Hafner<br>Victoria DeLeo<br>Cecilia H Deng<br>Christine G Elsik<br>Damarius Fleming<br>Peter W Harrison<br>Theodore S Kalbfleisch<br>Bruna Petry<br>Boas Pucker<br>Elsa H Quezada-Rodríguez<br>Christopher K Tuggle<br>James Koltes
**Order of Authors Secondary Information:**	
**Response to Reviewers:**	Dear Reviewers,<br>Thank you for your efforts to review and provide suggestions to improve our

Powered by Editorial Manager® and ProduXion Manager® from Aries Systems Corporation

manuscript. Please find point by point comments below for each suggest edit.

Best regards,
James Koltes and Alenka Haefner (for the authors)

Reviewer #1: The manuscript titled "Data reuse in agricultural genomics research: challenges and recommendations" provides an in-depth and timely analysis of the key challenges associated with data reuse in agricultural genomics, offering a set of actionable recommendations to improve these practices. The authors successfully identify and discuss critical obstacles such as data standards, metadata deficiencies, data interoperability, and equity issues, contributing valuable insights to the ongoing discourse in this area. While the manuscript is well-structured and presents a thorough examination of the subject, there are several areas that require further attention:

Major Points:

1. Lines 53-57: The manuscript references several major genomic data centers, but it omits the National Genomics Data Center (NGDC, https://ngdc.cncb.ac.cn/) and the Genome Sequence Archive (GSA, https://ngdc.cncb.ac.cn/gsa/). These should be included to provide a more comprehensive overview of available resources.

Thank you for your comment, we have now added the importance China National Center for Bioinformation's resources, including NGDc and GSA, to the international community.

2. Line 65: The examples of data reuse provided in the manuscript are helpful, but additional references would further strengthen this section. Consider including more examples from plant genomics, such as those found in these studies: 10.1093/nar/gkad706, 10.1093/nar/gkac930, and 10.1038/s41467-022-30770-1. The authors could explore more ...

Thank you for the additional examples of successful reuse. We have added plant examples that are agriculturally significant, including the references you provide. While two paragraphs in the Background section focus on these benefits, the main aim of our paper is not to argue the benefits of reuse as these are well-known and established in the field. That is why we refrain from expounding upon individual examples due previous reviews described in-depth the benefits of reuse (Sielemann et al., 2021).

3. While the manuscript covers a broad spectrum of challenges, certain sections would benefit from more in-depth analysis. The discussion on equity issues, for instance, is somewhat superficial. Expanding this section with detailed examples or case studies would provide a more robust understanding of the equity-related challenges in data reuse.

As we point out, the research on equity in big data has been very limited to date, particularly in the agricultural field. We have mentioned several studies addressing the issue (Harris et al., Xafis et al., Tiffin et al., Mott et al., Carroll et al.) and provided examples of how access issues can be addressed throughout this section. We find that the paragraphs on the issue of governance of Indigenous data is a very pertinent example and one applicable to many reuse cases a reader might encounter in agricultural genomics research.

4. The manuscript would be significantly strengthened by incorporating specific case studies or examples where data reuse has been successfully implemented in agricultural genomics. These practical illustrations would enhance the manuscript's applicability and offer readers tangible insights into the implementation of the recommendations.

Thank you for this constructive comment. While we have mentioned several examples of successful data reuse, we now expounded upon these in-text (lines 114-118 and 128-141). Please also see our response to comment no. 2.

5. The introduction effectively sets the stage by underscoring the importance of data

reuse in the context of open science. However, it would benefit from a brief historical overview of the evolution of data reuse practices, especially within the agricultural genomics domain. This context would help readers appreciate the current state of data reuse and the progress made thus far.

Thank you for this suggestion. We have edited the section on the history of data reuse practices to better describe how it pertains to agricultural genomics (lines 17-22) and direct the reader to an in-depth overview by Sielemann et al. (2021).

6. The discussion on metadata deficiencies is well-crafted, but it could be further enriched by addressing recent advancements in metadata standards. Discussing how these innovations could mitigate current challenges would provide a more balanced and forward-looking perspective.

Thank you, we have now added more examples of advancements in metadata standards in other big data communities (line 312), which provides an optimistic outlook for the agricultural community. We also highlight the new NCBI Datasets initiative (lines 328-332).

7. The recommendations offered are thorough and relevant, yet the manuscript would be more practical if it distinguished between short-term and long-term solutions. This distinction would help readers and stakeholders prioritize actions based on their available resources and infrastructure.

Thank you for your assessment of our recommendations for solutions. We have added several qualifiers throughout the manuscript to distinguish between short- and long-term solutions (lines 358, 366, 480, 503).

Reviewer #2: In this manuscript, the authors explored the challenges and recommendations regarding data reuse. It identifies limitations related to data standards, metadata deficiencies, data interoperability, data ownership, data availability, user skill levels, resource availability, and equity issues, and suggests solutions that relevant stakeholders can implement. Additionally, the article offers an optimistic outlook on the future of data reuse in genomics and transcriptomics.

Although the authors had concluded the current problems during the data usage, but the most important things were solved to these problems, they didn't give any solutions.

Thank you for your comment. However, we disagree that we did not provide any solutions to the problems we describe. The paper is structured based on providing many viable solutions to each of the problems we describe. These were also recognized by reviewer #1. As you mention, our aim was to provide and optimistic outlook which would not have been possible without providing practical recommendations for the community to solve the problems we identified. Each main-body section describing individual barriers to data reuse (Fig. 1) ends in a paragraph describing solutions to the barrier. Not all solutions apply to the data reuser – many have to be implemented by other stakeholders, e.g. publishers, which we have pointed out in the relevant sections.

Currently, we have different type of data, eg. RNA-data, Protein data, Metabolic data, Stero-seq data, with different type of data, we need individual solutions for each type.

We completely agree with the need to have data formatting solutions tailored for different data types. As sequence-based data is so common across so many fields and additional work is done to share this data, it makes sense to focus on these data where the biggest impact can be made, before tackling the more diverse and stratified data types. We agree that re-use of these other data types is important but will require additional considerations.

As elaborated in the Background, our paper focuses on genomic sequence data due to its ubiquity and importance to the agricultural community. Specifically, the manuscript states:

Powered by Editorial Manager® and ProduXion Manager® from Aries Systems Corporation

“While this perspective focuses on sequence- based data, it is important to acknowledge the issues facing phenotypic data reuse, particularly the prevalent ad hoc formats, lack of archives for storing and accessing data, and inability to share phenotype and genotype data together (due to agreements with industry or lack of infrastructure).”

The AgBioData initiative’s data reuse and other working groups are actively working on issues of other data types. While we mention other data types where pertinent, expounded in Future of data reuse is bright section, we consider in-depth analysis of them beyond the scope of this paper.

In the part of data formatting, the authors didn't clarify the other types of data not only for the normal genome data. I think they should add this part.

We are unclear on what you are referencing here and assume you are referring to the section entitled “Towards interoperability via data formatting”. Our response to the previous comment would also apply here - this review is focusing on sequence-based technology data reuse which still requires considerable forethought to improve data reuse.

In the manuscript, the author said that the process files need also should be upload, I think this can't be realistic. Researchers can upload the data, as well as with some script, otherwise the database need a large storage.

We believe you are is commenting on the passage below (lines 470 – 481). We believe this section honors and raises similar concerns as those expressed by the reviewer.

“Being able to demonstrate reproducibility in analysis is important, and too often proves impossible [87], but it is equally important that the datasets achieve their full utility potential through reuse for novel purposes [88]. The processes that are performed to analyze the raw data are often beyond the computational resources and skills available to most researchers who could benefit from them. Therefore, it may be useful to make processed data, such as transcriptome and genome sequence assemblies, genomic variants, and peaks identified using technologies like chromatin immunoprecipitation sequencing (ChIP-seq), available along with the underlying reads whenever possible. However, storing intermediates and final products of pipelines comes at the cost of increasing the amount of necessary disk space, an important trade-off to consider. A possible short-term solution to this bottleneck to reuse is to make all code used in the computational analysis available alongside raw and/or processed datasets.”

The intent in this portion of the manuscript was not to imply that all processed data should be made public and it is mentioned specifically in this passage that the computational demands would be too high to not prioritize what processed data should be shared.

However, our team argues that if intermediate processed files are important to the overall results or interpretation of the study, they should not only be made public, but be made machine readable and reusable (i.e., interoperable). For example, if a high-quality transcriptomes, catalogue of genome variants, or epigenetic variants are identified in a study, these results would serve as important resources to the scientific community. Thus, they would be valuable for reuse as support for new studies, or foundations for new research.

We believe that under FAIR standards, researchers and databases should strive for complete accessibility and reusability. That requires that, as you point out, at minimum all raw data and all used scripts must be made publicly available. However, even that does not guarantee reusability due to the variety of issues described in the paper. To combat that, we suggest some processed files should be made available whenever possible.

As you say, this is not always realistic, but we believe that it should be something that we strive for as a community to improve access. For example, researchers with poor computational resources may not be able to replicate the study to achieve the

	processed files.<br><br>Overall, I think this manuscript does not meet the magazine's requirements for innovation.<br><br>We believe the manuscript is innovative because, to our knowledge, this is the first attempt to bring perspectives of plant, animal, and microbial scientists, working on topics related to breeding, bioinformatics, physiology, genetics, etc. together. Such collaboration between agricultural communities in one forum has, to date, not been done to address data reuse.<br><br>Further, while reuse has been assessed (as cited in the paper), there have been no focused recommendations for different reuse stakeholders from experts with diverse scientific roles. Our team includes early-career and senior researchers from the public and private sectors. Collectively, we have experience from production to biocuration which allows us unique insight into all aspects of hands-on genomic data reuse. The paper also links to a living document on GitHub to provide data (re)users with recommendations to address the issues raised.<br><br>Therefore, in our view, we meet the criteria for GigaScience's review category, namely "systematic and substantial coverage of mature subjects, evaluations of progress in specified areas, and/or critical assessments of emerging technologies".
**Additional Information:**	
**Question**	**Response**
Are you submitting this manuscript to a special series or article collection?	No
**Experimental design and statistics**<br><br>Full details of the experimental design and statistical methods used should be given in the Methods section, as detailed in our Minimum Standards Reporting Checklist. Information essential to interpreting the data presented should be made available in the figure legends.<br><br>Have you included all the information requested in your manuscript?	Yes
**Resources**<br><br>A description of all resources used, including antibodies, cell lines, animals and software tools, with enough information to allow them to be uniquely identified, should be included in the Methods section. Authors are strongly encouraged to cite Research Resource Identifiers (RRIDs) for antibodies, model organisms and tools, where possible.	Yes

Have you included the information requested as detailed in our Minimum Standards Reporting Checklist?	
**Availability of data and materials**<br><br>All datasets and code on which the conclusions of the paper rely must be either included in your submission or deposited in publicly available repositories (where available and ethically appropriate), referencing such data using a unique identifier in the references and in the “Availability of Data and Materials” section of your manuscript.<br><br>Have you have met the above requirement as detailed in our Minimum Standards Reporting Checklist?	Yes

Powered by Editorial Manager® and ProduXion Manager® from Aries Systems Corporation

Manuscript

Click here to access/download;Manuscript;GigaScience_Data Reuse(Sept17).docx

Data reuse in agricultural genomics research: challenges and recommendations

Alenka Hafner[1,2]* (ahafner@psu.edu, https://orcid.org/0000-0003-4262-9176)

Victoria DeLeo[3] (toriedeleo@gmail.com, https://orcid.org/0000-0002-4315-8436)

Cecilia H. Deng[4] (Cecilia.Deng@plantandfood.co.nz, http://orcid.org/0000-0002-2954-762X)

Christine G. Elsik[5,6] (elsikc@missouri.edu, https://orcid.org/0000-0002-4248-7713)

Damarius Fleming[7] (damarius.fleming@usda.gov)

Peter W. Harrison[8] (peter@ebi.ac.uk, https://orcid.org/0000-0002-4007-2899)

Theodore S. Kalbfleisch[9] (ted.kalbfleisch@uky.edu, https://orcid.org/0000-0002-2370-8189)

Bruna Petry[10] (bpetry@iastate.edu, https://orcid.org/0000-0001-9559-8559)

Boas Pucker[11] (b.pucker@tu-braunschweig.de, https://orcid.org/0000-0002-3321-7471)

Elsa H. Quezada-Rodríguez[12,13] (qrelsa@comunidad.unam.mx, https://orcid.org/0000-0001-7789-4987)

Christopher K. Tuggle[10] (cktuggle@iastate.edu, https://orcid.org/0000-0002-4229-5316)

James E. Koltes[10]*(jekoltes@iastate.edu, https://orcid.org/0000-0003-1897-5685)

*Corresponding authors

[1] Department of Biology, Frear North, Pennsylvania State University, University Park, PA, US

[2] Intercollege Graduate Degree Program in Plant Biology, Pennsylvania State University, PA, US

[3] Bowery Farming, 10 Basin Drive, Kearny, NJ, US

[4] New Cultivar Innovation, The New Zealand Institute for Plant and Food Research Limited, NZ

[5] Division of Animal Sciences, University of Missouri; Division of Plant Science & Technology, University of Missouri, MO, US

[6] Institute for Data Science & Informatics, University of Missouri, MO, US

[7] Animal Parasitic Diseases Laboratory, United States Department of Agriculture Agricultural Research Service, Beltsville, MD, US

[8] European Molecular Biology Laboratory, European Bioinformatics Institute, Wellcome Genome Campus, Hinxton, Cambridge, Cambridgeshire, UK

[9] Department of Veterinary Science, Martin-Gatton College of Agriculture, Food, and Environment, University of Kentucky, Lexington, KY, US

[10] Department of Animal Science, Iowa State University, IA, US

[11] Institute of Plant Biology & BRICS, TU Braunschweig, Braunschweig, Germany

[12] Departamento de Producción Agrícola y Animal, Universidad Autónoma Metropolitana-Xochimilco, Ciudad de México, México

[13] Centro de Ciencias de la Complejidad, Universidad Nacional Autónoma de México, Ciudad de México, México

Abstract

The scientific community has long benefited from the opportunities provided by data reuse. Recognizing the need to identify the challenges and bottlenecks to reuse in the agricultural research community and propose solutions for them, the data reuse working group was started within the AgBioData consortium framework. Here, we identify the limitations of data standards, metadata deficiencies, data interoperability, data ownership, data availability, user skill level, resource availability, and equity issues, with a specific focus on agricultural genomics research. We propose possible solutions stakeholders could implement to mitigate and overcome these challenges and provide an optimistic perspective on the future of genomics and transcriptomics data reuse.

Keywords: data reuse, metadata, big data, genomics, transcriptomics, agriculture, data standards, FAIR

Background

The value of data reuse is one of the founding postulates behind the Open Science movement yet remains an under-examined aspect of researchers' experience of open data [1]. Historically, global sharing of biological datasets became technically possible with the rise in access to the World Wide Web, and data reuse transitioned into an attractive option for researchers through benefits that came with an increasing number of available datasets and reuse applications, as reviewed by Sielemann et al. [2] In the past three decades, agricultural researchers have also adopted data sharing and reusing practices due to these benefits alongside the rest of the genomics community. Genomics data is particularly amenable to reuse, as many different types of structural and functional data are provided as DNA sequences, and many analytical tools have been developed to analyze and integrate genomics data types [3]. With constantly emerging sequence-based technologies, the language of nucleotides has become increasingly ubiquitous and useful. Alternatives for assays that traditionally have generated difficult-to-share data types, such as flow cytometry fluorescence, yield easy-to-share sequence-based data types to directly integrate RNA and protein modalities [4]. However, no dataset is perfect and data producers can only strive to satisfy the requirements for its initial use and reuse. Some researchers have identified the risks and challenges associated with data reuse in the life sciences[5,6], which informs agricultural data management [7], but a detailed assessment of the reuse issue in this area has not been conducted yet.

Recently, a report on the status of open data called attention to the importance of data availability in reuse [1]; however, barriers remain in making data amenable for reuse. Our objective in this perspective is to highlight concerns in data reuse across the agricultural genomics community to identify major challenges and viable solutions. We also provide our perspectives on best practices for sharing data to make it more accessible and reusable, as well as how to reuse publicly available data.

We define data reuse as the practice of utilizing existing data for a novel scientific purpose beyond its original scope. Although we recognize that this definition would include the use of reference genome sequences, we find that their reuse comes with unique challenges beyond the scope of this paper. Furthermore, while the reuse of one's own data fits under our definition, the recommendations and perspectives set out in this paper apply primarily to data reuse by researchers other than the data producer's group.

While types of data in agricultural research are diverse and go beyond sequence-based datasets, the sequencing community harbors a long-standing tradition of data sharing. A major advantage of genomics data for agriculture is that most of such data has a common sequence format and ontology, allowing the reuse and tuning of tools developed in the well-funded biomedical sphere. Reuse in genomics research is largely facilitated by the International Nucleotide Sequence Database Collaboration (INSDC) [8]. The INSDC consists of the National Center for Biotechnology Information (NCBI), The European Bioinformatics Institute (EMBL-EBI), and the DNA Data Bank of Japan

(DDBJ), which collectively support the Sequence Read Archive (SRA) and the European Nucleotide Archive (ENA). Additionally, the China National Center for Bioinformation's (CNCB) National Genomics Data Center and its Genome Sequence Archive also support the international genomics community [9]. Due to its predominance, we will focus on the reuse of sequencing data in this paper, while acknowledging the importance of other data types and emerging analysis technologies in the reuse research arena.

Reusing existing data brings significant benefits for scientific research, such as saving time and cost without generating new datasets, enabling meta-analyses and interdisciplinary research by combining data from multiple studies, or new discoveries by exploring novel hypotheses through integrating data from different sources or using innovative analytical techniques. More and more exciting publications are being produced that highlight the value of data reuse, both in the animal and plant side of agriculturally-important research [10–17]. Still, many datasets are not reusable, or scientists may feel they do not trust or do not want to use the data [18]. Several review articles have discussed the opportunities and challenges of data reuse [5,19–22], the latter highlighted in Figure 1.

Figure 1. Biological data types are diverse, and their reuse comes with unique challenges. The barriers and limitations of data reuse discussed here include data quality and standards, missing metadata, issues of formatting and interoperability, lack

of data availability, ownership, and intellectual property, and access to resources and skills.

Principles of Findability, Accessibility, Interoperability, and Reusability (FAIR) are essential to enable successful sharing and reuse of datasets in the ‘Big Data’ world [23]. The science community has also agreed to uphold data sharing practices that enable data reuse through accords and requirements that promote it [24–28]. Recognizing the value of reusable datasets and the ubiquity of FAIR principles might lead one to believe they are universally accepted and applied. However, as any data stakeholder can testify, no dataset is without flaws[6] and a multitude of problems can present themselves to a potential re-user.

Once initial challenges to sharing are overcome, the reuse of existing datasets has numerous advantages [5]. Designing experiments, collecting samples, and generating data usually involve extensive time, effort, and funding. Retrieving datasets from a repository and reusing them speeds up the research as the analysis can be started immediately. Biologists can generate new hypotheses to inform their experiments or analyze existing data for preliminary results for emerging research proposals. Alternatively, they may analyze public datasets as additional evidence to test hypotheses in their studies. Through the reuse of datasets from public domains, it is possible to investigate massive datasets for data-driven discovery that would not be viable to generate as part of an individual study or explore datasets of species that would not otherwise be accessible. Examples include datasets that were compiled over

multiple years and represent a substantial number of species in a certain taxonomic group, e.g. Earth BioGenome Project [29] and Vertebrate Genomes Project [30]. Finally, reused datasets enhance the equity of science as they are available without substantial costs and allow anyone with sufficient computational resources to benefit from cost-effective data sharing, contributing to the inclusion of early-career and underrepresented scientists [5]. Bioinformatic software developers can rely on publicly available datasets for their benchmarking studies, making it possible to evaluate the performance of novel bioinformatic tools based on real datasets. Biologists can perform analyses to generate hypotheses to inform their experiments or include public datasets as additional evidence in their studies. As demonstrated in the agricultural field by the CattleGTEx atlas [31], the power of data reuse is growing with emerging technologies and the integration of enormous amounts of data. This includes harnessing high-quality datasets for analysis using machine learning and cloud computing, for example the analysis of over 3000 rice genomes by *DeepVarant* [32], as well as using real datasets as quality control for synthetic and artificial intelligence-generated datasets. The benefits of shared infrastructure and avoidance of resource multiplicity, as embodied in NSF’s Synthesis Centers [33], enable productive and efficient investigations into new questions using ‘old’ data, a desirable future for agricultural research.

A unifying objective across biology is understanding the link from genome to phenotype (G2P) to move toward predictive biology; reuse of existing datasets will play an important role in this process. G2P initiatives both depend on and act as a test of, existing data reuse standards and infrastructure. In this way, G2P will also identify

where deficiencies exist in data reuse resources. Different funding organizations fund these long-term goals through requests for applications (RFAs). For example, the Genome to Phenome Blueprint [34] discusses the importance of data reuse for animal genetics as a 10-year research priority as identified by researchers at the United States Department of Agriculture (USDA), also reflected in their *Agricultural Genomes to Phenomes Initiative* [35–37], while the National Science Foundation (NSF) runs the *Understanding the Rules of Life* program [38]. These RFAs all seek ways to improve data reuse as it is believed that integration of data across diverse and expansive datatypes is needed to identify novel phenomena regarding genome function. Tuggle et al. describe the shared efforts of the animal and plant genomics communities to develop synergies and leverage strengths to advance genome-to-phenome research to make scientific advancements that will accelerate applications in agriculture to help feed a growing world under a variety of challenges [35,36]. Comparative and evolutionary biology studies [29,30,39–42] are also important initiatives whose data will need to be amenable to integration and reuse to help in these efforts. While this perspective focuses on sequence-based data, it is important to acknowledge the issues facing phenotypic data reuse, particularly the prevalent *ad hoc* formats, lack of archives for storing and accessing data, and inability to share phenotype and genotype data together (due to agreements with industry or lack of infrastructure). For G2P initiatives to be successful, sequence-based and phenotype datasets need to be combined, overcoming their respective barriers to reuse and challenges of integration.

To assess the data reuse needs and obstacles that this community faces, our working group explored the challenges associated with data reuse (and their potential solutions) through personal testimonies and discussions within the AgBioData consortium's Data Reuse Working Group (DRWG), as well as a review of pertinent literature. The DRWG represents a diverse group of researchers with varied interests in species and scientific applications of data within the domain of agriculture. The AgBioData Consortium [43] is a group of genomics, genetics, and breeding databases and partners working to consolidate data standards and best practices [44–46]. The issues and opportunities presented here were generated as part of regular meetings, conference presentations, and workshops held as part of a data reuse project funded by the USDA AG2PI [37].

Barriers to data reuse and recommendations to overcome them

Consider a potential data re-user in agricultural research on their path to a dataset, as depicted in Figure 2. They are seeking data from an experiment they learned about at a conference and can locate the paper in which the dataset was originally used. Sometimes they need to email the corresponding author to overcome the broken link to the datasets, and they eventually find the dataset in an online repository (if the data are not in local storage instead) (Figure 1A). The dataset itself might be of unknown or poor quality, from undisclosed provenance, without proper documentation, or contain incomplete or even incorrect metadata. All these factors can generate confusion in the comprehension of the data and make their reuse challenging. Our re-user must assess

whether their subjective requirements of “quality” are met before deciding to reuse the dataset (Figure 2B). Data ownership rights must be checked and can be difficult to adhere to with older, missing, or ambiguous licenses. The next problem the re-user might encounter is the format of the dataset and if it can be correctly and successfully interchanged into a configuration their downstream analysis supports, which might depend on their skill level (Figure 2C). If they are attempting to retrieve large datasets from a study, they might not have access to sufficient computational resources to store the raw datasets or run the analysis (Figure 2D). The intermediate results produced in the original study, which could partially remedy the storage problem, may not be available on the repository. It is also likely that intermediate results were produced based on an outdated version of the reference genome sequence or its annotation. Furthermore, the hopeful re-user could be a student, who seeks counsel from their advisor but is informed that the experiment (or public data in general) is untrustworthy, or unsuitable, because of ethics or proprietary constraints. For reuse of a dataset to be successful, these issues must be overcome. The prevalence of these problems can vary depending on the data type, prominence of the original study, the repository they are in, and user skills. However, most stakeholders acknowledge that these issues remain problematic [18], including in agricultural research.

Figure 2. Workflow chart depicting potential pitfalls preventing data from being reused. Bolded lines follow the minimum number of steps/questions a potential re-user needs to consider. Dashed red lines denote steps that lead to a dataset not being reused due to circumstances that do not have to do with the qualities of the dataset

itself. Green and red lines lead to outcomes of data reuse after a critical question in dataset assessment is answered yes or no, respectively. The workflow is divided into two parts (blue line) based on the FAIR principles of a dataset being findable and accessible, while also interoperable and reusable. A, B, C, and D denote major decisions or workflow divergence points.

Data quality standards as a solution

No dataset is perfect [5,6], but that does not mean it is not suitable for reuse. As data are made publicly available regardless of the quality metrics, data quality assessment and standardization are important considerations [6] (Figure 2B). Statisticians are well aware of this issue [47], which is particularly problematic in the life sciences likely due to the complexity of biological systems, number of variables, and scale of experiments. The difficulty in obtaining and understanding the context surrounding the available data has been identified as a major obstacle to reuse in synthetic biology [48] where interdisciplinarity is one of the defining features of the field. We can extrapolate similar issues to agricultural research, which often involves cross-disciplinary collaboration that combines diverse (meta)data types requiring integration and analysis.

To assess if and how a publicly available dataset can be used in analyses beyond its original purpose, a decision must be made about whether it is suitable for reuse. In a sequence-based context, data suitability can mean a variety of dataset properties, including coverage, depth, technical and biological replication, tissue type and sample collection method, extraction method and library preparation, and other criteria. Further,

sequencing technology, platform, chemistry kits used, flowcell version, and related information must be considered as is required by basecallers for conversion into the sequence. All these technologies are also continuously under fast-paced development. With this in mind, whether a dataset is of sufficient quality and suitable to be reused is a difficult, and largely subjective decision [49] and varies between applications. While there are some data type-specific standards available (e.g., *Genomic Data Commons* [50]), their scope is limited. Agricultural research is often multidisciplinary, has complex experimental designs, and spans many non-model species, which makes applying any universal standard very difficult.

Unified experimental protocols or bioinformatic pipelines for common data types and organisms are rare. This is not a problem in and of itself at the level of data production, although an off-the-shelf pipeline could streamline the process and provide benchmarking for workflow development. The lack of standard protocols and pipelines is problematic when it comes to data reuse. Not only can it be difficult to obtain the exact experimental protocol used (e.g. discussions of data reuse often result in anecdotes of lost protocols with unanswered emails and/or students who graduated), but meta-analyses are also hindered by a lack of standardization. Sharing experimental designs and protocols together with produced datasets is a challenge that the international data standards rarely address. Examples of minimum information standards being implemented by necessity include the Minimum Information About a Microarray Experiment (MIAME) and Minimum Information about a Sequencing Experiment (MINSEQE) [51].

Further, an important question that needs to be considered in the field is whether our experiments should be designed with future data reuse in mind. For example, while for the original data producer, one biological replicate may have been sufficient for the purposes of gene prediction, a statistically robust meta-analysis of gene expression may require at least three [52]. Such meta-analyses must solve the important issue of handling batch effects when merging data from multiple sources and attempting to use multi-source replication for statistical analysis. Not only can the complete datasets be harnessed in the future, but they can also limit the need for the same sample to be sequenced again, saving resources for dataset production and storage. However, upfront costs of production are shouldered by the original data producer and prohibit much consideration of potential future reuse benefits. A model for partially transferring the costs of the initial experiment from the individual to the community would be required as an incentive for additional data generation. Additionally, future use objectives can be difficult to predict, and emerging technologies can make numerous datasets irrelevant. The most important step that can be made by the data producers, journals, and funding agencies in ensuring future reuse is to submit complete metainformation, including recorded factors that were not relevant to the original study.

Looking at the example of the biomedical sphere in solving issues of data quality, the agricultural research community should adopt more standardization across the board. While file type standardization is common for sequence-based data (e.g., FASTA or FASTQ), there is a lack of experimental protocol, sample handling, computational

pipeline, and statistical standards present in agricultural research. This makes assessing data quality one of the biggest barriers to dataset reuse. Unified recommendations, if not standards, for all aspects of data collection, would enable more successful data reuse, increasing a dataset’s economic utility, with the added benefit of aiding the data producer in making their research more broadly comparable. The AgBioData Genome Nomenclature working group is currently trying to address this issue. Such standards need to be broadly applicable and not too severe, in a “legacy standard” format that does not hold back future stricter requirements and developments in the field.

Incentivizing complete metadata for reuse

The missing information about datasets available to a potential re-user exacerbates the problem of lacking metadata standards. Historically, the need for minimum metadata standards was recognized and implemented by many journals and funding agencies, but missing metadata is still one of the main barriers to data reuse cited by researchers [5,49].

While most sequencing datasets are released through INSDCs databases [44], there is a sparsity of metadata accompanying them. For example, the precise tissue type, cultivation conditions, or developmental stage may not have been recorded. Complete metadata is especially important for RNA-seq datasets because the transcriptome responds quickly to the environmental conditions of the sampled individual. As DNA methylation can now be investigated based on Oxford Nanopore Technologies or

Pacific Biosciences HiFi sequencing data, information about the conditions prior to DNA extraction gains importance. Re-users might want to study the methylation of DNA in response to certain environmental conditions or treatments. Further, methods used to minimize sample-to-sample variation due to sequencing methods, such as barcoding of pooled samples, must be clearly explained. If there is data from the same sample sequenced in different lanes to increase the sequencing coverage, this needs to be annotated in the metadata table, as it can lead to confusion when distinguishing samples that were just sequenced in different lanes from replicates.

The paradigm of ontologies has enabled the interoperability and reuse of data in the genomics era [44,53,54]. However, using available ontologies to describe data from agriculturally relevant species is often not appropriate, as such tools are model organism- and medical-based. Initiatives like the *Genomic Data Commons* [50] do provide scaffolds of metadata standards but are limited to a small number of data types and purposes. Furthermore, metadata submission templates tend to only work for some organisms or sample types, and do not enforce the use of controlled vocabularies. Smaller, community-based efforts are on the way to improve available ontologies (e.g., MIAPPE [11] and FAANG's *Ontology Improver* [55]).

The biggest effort to integrate data and metadata with available controlled vocabulary standards is the INSDC [56]. It enables extensive data sharing and interoperability, with the responsibility for the quality and accuracy of the record naturally falling on the submitting author, not on the database [57]. Interoperability standards in medicine for

genotypic and phenotypic patient data [58] could be informative for agricultural research as well. These health information formats include metadata on the tests run, and sometimes even on the analyses not run, to enable healthcare providers to integrate results from diverse panels. Such complete metadata could generate a large overhead in some circumstances and must be considered in the context of agricultural genomics. Various communities have proposed guidelines for standardizing metadata [59–62] and minimum information standards in experiments (MIAME and MINSEQE), but there is still a need for more comprehensive standardization of metadata across different databases, both in what is captured and how it is captured.

Without incentives or requirements, researchers often seek the lowest effort route to publication with minimal metadata. As the submission of metadata can require substantial work, there is a trade-off between collecting all datasets via a lenient submission system and mandating comprehensive metadata to boost the reuse potential of datasets [5]. Initiatives like *nfdi4plants* [63] in Germany are working to make data submission as convenient as possible. Ideally, submitting users would be supported by automatic completion of certain fields. Data documentation takes extra effort, necessitating the need for a reward system to encourage the production of datasets amenable to reuse. This could include dataset citations, credit for shared data in promotion, and other rewards for datasets that are reused often and successfully.

A major step forward is the recent launch of NCBI' Datasets resource which is guided by FAIR principles and delivers, among other tools, simplified discovery and access to

metadata [64]. One of the motivations behind the initiative is that "*explicit linkage between sequence data and its metadata facilitates improved reusability and proper attribution*" [64].

Towards interoperability via data formatting

The genetics and genomics community converged rapidly on data format standards and is on the road to establishing standards for the metadata stored within data files [46,65]. Widespread standardization of these file formats facilitates easy interconversion and use by analysis and visualization software, ensuring interoperability. The Sequence Alignment Map (SAM) format for high throughput sequence data, and its respective mapping results, requires the recording of a data dictionary with information on the reference genome sequence used for mapping, such that can ensure any subsequent analysis will be required to use the same reference [66]. There are also provisions therein to record data processing information, such as the program and command line used to generate the mapped dataset and any post-processing, including sorting and PCR duplicate removal. Other standardized formats with enforced rules include the SAM compressed format Binary Alignment Map (BAM) [67], the Variant Call Format (VCF) [68,69], the Gene Transfer Format (GTF) [70], General Feature Format (GFF3) [71] and Browser Extensible Data (BED) [72] files that allow for annotation of regions of a given genome sequence [73]. All these files can be coordinate indexed such that they may be searched, and subset easily by locus or loci.

As evidenced by the wide acceptance of universal data formats in genomics research, the limitation to the wider adoption of data reuse is not the lack of defined data formats, but the consistency of their use. Many datasets are deposited according to the parameters of the database chosen to hold the data. The database may allow for several types of files when it comes to, for example, transcriptomic studies. A researcher has the option of uploading the data in the form of a set of FASTQ files or maybe as a set of BAM files, with the choice made dictating how reusable the data can be for others. A possible short-term solution is for the repositories to provide more re-user-friendly tools that facilitate interconversion between formats, for example, FASTQ and BAM, without accompanying loss of metadata.

Although the genomics datasets of types mentioned above have documented standards requiring information such as what reference genome sequence and what version were used for their analysis (standards enforced by assertions in analysis packages like the *Genome Analysis Toolkit* [74]), mapping to reference genome sequences does create an impediment to interoperability with processed, or secondary datasets. Any long-term solution to this problem would require reference-free analysis of data. This is an area of active research [75–77], and a future in which indices accompany raw datasets for rapid query and use in synchronous analyses that run at remote sites seems possible. Further, data types not based in genomic sequence, for example proteomics and metabolomics, require their own standardized formatting and face unique issues of reference-gated interoperability [78,79].

Interoperability with data from outdated wet lab and/or computational analysis methods can also present a challenge. A few tools have been built to bridge the data found in newer, standardized sequencing files with data encoded by older formats such as arrays and spa typing [80–82]. To guard against data obsolescence, researchers need to incorporate thorough analysis workflows (for example, using resources like Protocols.io [83] to enrich metadata for methodological detail. Hence, interoperability is also supported by adherence to metadata and data quality standards described in previous sections.

To encourage interoperability, data warehouses, and journals can raise their standards for data submission to require the inclusion of the outputs of primary analyses. This practice is often encouraged, but not required or enforced. Synthesis Centers (funded by the NSF) are examples of projects that highly promote data reuse and integration and reuse are ubiquitous, demonstrating the economic efficiency of data exchange with incredible success [33]. Recent efforts have also been made to boost interoperability in the Bgee knowledge base by taking stock of file-based data exchange, programmatic interfaces, and automatic interoperability efforts [84]. The good news is that interoperability boosting seems to have a positive domino effect enabled by automation, which will hopefully lead to near-total integration capabilities soon [84], although benefits perceived by all stakeholders are still lacking [54].

Bridging the data availability gap: a role for all stakeholders

A major barrier to reuse is the availability of data with their accompanying metadata and sample information in repositories (Figure 2A). It is crucial for data providers to include all samples and relevant information in a clear sequence, using the provided data format or metadata template when available. This includes raw data and metadata, including sequencing methods, sample name, tissue, organism, project, and associated papers. The information provided needs to be clear and comprehensive to facilitate the reproducibility of analyses. The commitment of all data stakeholders is crucial in narrowing the data availability gap as summarized in Figure 3.

Figure 3. Recommendations for bridging the data availability gap include data producers, scientific journal publishers, and funding bodies as stakeholders.

Many journals provide generic statements for authors to declare that all data are included in the supplementary files of the article or deposited in a public repository. However, such statements are not helpful without specific accessions or links that point readers to the respective datasets. A further contributor to this data availability gap is the “data available on request” statement present in many papers that do not provide a direct link to their data in a repository but ask the potential reuser to contact them to receive it. A study on data availability from papers published in *Science* and *Nature* in 2021 found that an alarming less than 50% of data stated to be “available upon request” could be effectively obtained from the original authors [85]. Further, about 20% of all metagenome assemblies are not easily accessible due to the lack of accession

numbers in the publication or due to empty accession numbers [86]. Even if data are provided, it can take months to receive it [85], with questions about storage and management arising. More encouragingly, after many attempts at contact, 83% of data was made available at least partially [85].

Journals could improve the situation by providing more detailed templates that require researchers to fill in accessions or URLs and to include data accessibility as a criterion for reviewers to assess. Options to link a GitHub repository with code, Open Science Framework material, or specific datasets to the submission would be another option. However, enforcing such data standards requires additional labor by editorial staff and reviewers. While journals would be well placed to enforce a policy that would benefit reuse, funding bodies could be in an even stronger position to mandate rapid publication of all datasets under an open license. Data management plans are required parts of grant proposals but are not enforced or checked for compliance in subsequent applications. Automatic checks of the submitted datasets would be helpful to reduce the amount of work that reviewers need to invest in the technical aspects of a journal article or grant proposal submission.

Datasets should be shared through the repository appropriate for the data type as summarized by Deng et al. (Table 1) [44]. For example, RNA-seq datasets should be submitted to Gene Expression Omnibus (GEO) to make precomputed count tables and the underlying raw sequence reads available. The reads are passed on to the SRA which also mirrors them through the ENA and the DDBJ. This ensures the preservation

of the data. Direct submission of RNA-seq datasets to the SRA/ENA/DDBJ is possible and common but does not allow the sharing of already computed count tables. This places a burden on researchers trying to reuse these datasets. Genomic sequencing data are best placed in this mirrored database to ensure availability to the community. Accession numbers for data submitted to repositories should also be included in publications. Generalized repositories often have minimal metadata requirements that suit many data types and support open data, but do not enable FAIR use. More specialized databases that serve specialized communities can often be better suited for detailed metadata sharing and can be contacted by authors for advice. As more data management plans contain a machine-readable requirement, direct collaboration with repositories becomes even more important.

All data published to sequence archives are data that have had some primary analyses, including quality control, performed on them. For next-generation sequence data, nearly all will have been mapped to a reference genome sequence. Whole genome shotgun sequence data will likely have been variant called and will have, at least a VCF file, in addition to the BAM file and the mapped FASTQ file. RNA-seq and epigenetic datasets will have been mapped, and likely have quantified transcripts and peak sizes respectively. For example, DNA methylation data will often supply only raw reads in FASTQ and differentially methylated regions, the latter representing the final output of highly variable and long pipelines. For the most part, the data that are being stored and are filling up public repositories are the raw FASTQ files. Due to the large sets of information and calculations needed to examine all manner of “omics” data,

computational methods are employed for analyses. In some cases, the analyses require the authors to write code, yet they often do not share the code itself, diminishing the usefulness of the shared data.

For such datasets to be reused, scientists are required to not only download the raw data but also reprocess them. This re-analysis is likely to generate many identical pipeline intermediates and final datasets that were created by the original analysis. Being able to demonstrate reproducibility in analysis is important, and too often proves impossible [87], but it is equally important that the datasets achieve their full utility potential through reuse for novel purposes [88]. The processes that are performed to analyze the raw data are often beyond the computational resources and skills available to most researchers who could benefit from them. Therefore, it may be useful to make processed data, such as transcriptome and genome sequence assemblies, genomic variants, and peaks identified using technologies like chromatin immunoprecipitation sequencing (ChIP-seq), available along with the underlying reads whenever possible. However, storing intermediates and final products of pipelines comes at the cost of increasing the amount of necessary disk space, an important trade-off to consider. A possible short-term solution to this bottleneck to reuse is to make all code used in the computational analysis available alongside raw and/or processed datasets.

Sustainably storing ever-growing datasets is a current and growing challenge. Disk space and electric power consumption will continue to rise as database sizes increase and data reuse becomes more popular at research institutes and companies. There is a

recent trend to move analyses to the data instead of moving the data, for example through cloud computing[89]. Given the explosion in dataset sizes, this seems like a logical step to take, since many large datasets are already available within a cloud environment. However, this harbors the risk that datasets will be effectively locked behind paywalls, as users would be required to pay for the computational resources. Once fully established, such a system could lead to expensive charges beyond the costs of maintaining the cloud infrastructure. It would be important to have a publicly funded infrastructure or to ensure sufficient competition between several providers. Efforts for establishing more sustainable funding of biodata resources are already underway (e.g., the Global Biodata Coalition [90]) as are community recommendations for sustainable database management[91].

As citations of scientific publications are considered the currency of science, citations of datasets could acquire similar importance [92]. Open Science Framework [93] provides scientists with options to easily share datasets that are citable and searchable through Digital Object Identifiers (DOIs). The benefits associated with the publication of paper preprints extend to datasets mentioned in them, enabling instant dissemination and citation of DOIs. A cultural shift or requirement is needed in the long-term to ensure that dataset identifiers are included in the main text of publications, enabling automatic readers to discover them. Additionally, automated literature tracking solutions could credit the impact of a dataset, by tracking whenever this dataset is mentioned in a subsequent publication (e.g., DataCite [94]). For meta-analyses that contain large numbers of datasets that cannot all be mentioned in-text, it would be necessary to

develop an automatic screen that searches all supplementary files for mentioned DOIs. Such a screen could be extended to patents to analyze the commercial relevance of datasets.

Rewards for well-documented data submissions could be a strategy to further improve the quality and quantity of publicly available datasets [95]. Among them could be an evaluation criterion for research proposals of data an investigator has shared in accordance with data sharing plans in previously funded research projects. Researchers spend substantial amounts of time and resources on generating and submitting datasets. This could be rewarded by tracking the number of studies re-using these datasets, as attempted by the Omics Discovery Index (OmicsDI)[96]. Funding agencies, universities, and companies would need to make hiring decisions based on this criterion, similarly to how they already do with publication citations. As this would be a rearward-facing statistic, it would likely come with the same biases and issues of equity as citations of scientific publications, namely self-citation, gender, racial, and institutional bias[97], but may still incentivize the generation of more reusable datasets.

Data ownership and sharing requirements

An important source of genetic material for research in plant and animal genomics is samples from genetic lines derived from breeding companies that have current commercial value or intellectual property. Often, arrangements to use such data for experiments are important for omics analyses to be relevant to species of agricultural importance. Breeding companies often have large populations with excellent metadata

and can provide samples at little to no additional cost. However, these companies need to protect their investments in intellectual property and often prohibit researchers from making their sequence or omics data public (e.g., a recent dispute over intellectual property rights for improved seeds [98]). Unfortunately, this is a major barrier to reusing relevant agricultural data.

There is a challenge in having access to relevant, affordable study populations from breeding companies that can also be shared publicly as sequence or genotype data. The extent of sharing is also unknown as a reliable assessment of the economic importance of datasets would be difficult to achieve because most companies could not permit an analysis of internal data reuse to protect their intellectual property. Enabling a self-reporting system could be an approach to gain insights into data reuse within companies, in addition to the dataset citation reward system mentioned in the previous section. Finding common ground in pre-competitive research spaces and ways to leverage industry data for scientific discovery, while protecting intellectual property, will help facilitate the reuse of some industry data.

Maintaining the competitive value of industry data is important, thus, there is a need to develop novel data-sharing solutions that protect intellectual property but facilitate more data sharing. Several methods have been proposed to overcome this problem, including homomorphic/monomorphic encryption and federated learning methods [99–102]. The inability to share industry data inhibits publication in an increasing number of journals. Additionally, it also threatens to reduce public-private research partnerships funded by

the US government as pending regulations will require all data funded by federal grants to be made public tentatively sometime in 2026 [103].

Agricultural industry datasets provide value to both the public and private sectors and importantly facilitate innovative training of graduate students. The ability to reuse industry data impacts graduate student training since students are required to produce publications and demonstrate competency based on their expertise. Reduced access to industry data will diminish training sought by industry to work with industry-relevant data. Thus, challenges related to data reuse of industry data have a broad impact.

Another consideration is data generated from biological resources that are maintained by specific cultural groups (discussed below in *The importance and benefits of equity and inclusion in agricultural data reuse*). Landraces, traditional crops, and crop wild relatives contain valuable genetic variation. There are weak systems in place to guarantee the engagement of these communities when their data is used and reused [104,105]. The human genomics community has experience in data privacy to maintain HIPAA compliance to ensure healthcare data remains both private and portable. The use of data management, sharing, and processing tools developed for medical systems may help overcome some of these challenges in agriculture.

There already exist numerous federal grant data sharing requirements. Genetic sequence data is an increasingly important consideration in policy regarding agricultural intellectual property rights and conservation (e.g., The Nagoya Protocol [106],

International Treaty on Plant Genetic Resources for Food and Agriculture [107], African BioGenome Project [108]). The upcoming 2026 mandate to make research funded by the US government publicly available [103] will undoubtedly alter the landscape of data sharing and ownership further. When it comes to future publicly funded research, we believe that partnerships between public and private entities should prioritize collective benefits to ensure that the rewards of data reuse are reaped equitably.

Resource availability and user skill level

Concerning high throughput sequence data, the data that are stored are typically unprocessed sequence datasets in FASTQ format. For most genetic or genomic studies, this format is the starting point for any analytical pipeline. The bioinformatics skills and computational resources required to store and transform FASTQ data into, for example, quantified expression levels, variants, or genotypes, exist in most larger research institutes. Therefore, we believe that many issues of data storage and computational resource availability are not the limiting factors in most US-based academic and government institutions any longer (which could be said a decade ago) (Figure 2A, C, D). However, worldwide many agricultural researchers and institutions do not have ready access to these resources. This constitutes a barrier to the reuse of these data, which for many, is insurmountable, constituting a major challenge to equity and inclusion in the future of data reuse.

Additionally, user skill level, awareness of resources, and time investment into data management are likely inhibiting a lot of productive reuses and limiting how many

resources are being made available for future reuse (Figure 2D). A recent study [20] shows that, at least anecdotally, skill or perceived ability was identified by many participants as a major factor influencing reuse behavior. Concerning methods of data storage, sharing, and management were identified across all science sectors and types of research activities, with most respondents to a 2017-2018 global survey of scientists exhibiting “high and mediocre risk data practices", for example storing data on USB drives [18]. That same survey found that attitudes toward data reuse were mostly positive, but that practice does not always support data storage, sharing, and future reuse [18]. Investment into data literacy early in science education will address these issues in future generations of researchers[109]. We agree with Tenopir et al. [18], namely that “*programs for both awareness and to help engender good data practices are clearly needed*”. Further, data reuse can be incentivized using award systems for successful reuse cases, for example, the DataWorks! Prize [110] or The Research Parasite Award [111].

The importance and benefits of equity and inclusion

The introduction of Big Data in agriculture has provided tremendous opportunities for advancements [112]. Equity considerations are essential to ensure that the benefits of agricultural data reuse are shared equitably among diverse stakeholders, including marginalized communities and vulnerable populations [113].

The reuse of data can improve equity and inclusion by reducing costs and increasing dataset utility. Nonetheless, the reuse of data requires computational capacity, internet

access, digital literacy, and proficiency in dominant languages. Despite significant global disparities, nations are formulating policies and expanding infrastructure to reach remote, rural, and peri-urban communities. The percentage of people with internet access has been steadily increasing, although each locality has its own unique needs. The internet plays a pivotal role in bridging the gap to access a wealth of information.

The knowledge disparities can be narrowed by employing data visualization techniques and providing commentaries, detailed explanations, glossaries, and links to both basic and complex information. Data visualization, defined as “information which has been abstracted in some schematic form, including attributes or variables for the units of information” plays a pivotal role in assisting non-data scientists in comprehending and effectively reusing data [114]. In contemporary data science, professionals are increasingly incorporating advanced technologies into data visualization, including algorithms, human perception, animation, and the development of computer graphics and software. These innovations enable the discovery of valuable insights within vast datasets [115].

Documentation of data is essential for facilitating reuse, and it is crucial to link the outcomes of data reuse with contextual information. Scientists require technical details regarding equipment and data procedures, maintenance of data formats, ontologies, and metadata within a specific field [116]. However, individuals with varying levels of knowledge disparity often need access to more information. To address this need, databases and repositories for reused data should be linked with institutional science

communication websites, providing comprehensive explanations of fundamental concepts.

Equally, as numerous studies have shown, diversity breeds innovation [117] (Figure 4). Thus, to harness the full power of a data-driven future in agriculture, the omics community needs to wrestle with the question of whether biases present in research citation patterns (prestige of the authors being cited, their gender, race, and nationality [97] are transferred to datasets which are selected for reuse.

Figure 4. Data reuse can facilitate a positive feedback loop between striving for diversity, equity, and inclusion, and the benefits of big data in agricultural research. This may include capturing more diverse and creative solutions to problems and diversifying the agricultural genomics community.

It is also vital we adhere to and enforce the CARE (Collective Benefit, Authority to Control, Responsibility, and Ethics) principles for Indigenous data governance [118] of existing and future datasets. As Carroll et al. [118] note, we must acknowledge that many publicly available and reused datasets already use Indigenous resources and traditional knowledge. A great resource for data sovereignty-enhancing research is the Local Contexts initiative [119], providing "*a digital infrastructure for community governance of Indigenous data*". Our recommendation to the community is to engage with Indigenous communities, practice responsible data stewardship, and use Indigenous ethics to determine data access [120]. This includes the use of appropriate

digital identifiers and inquiry into and respect for ownership rights. Traditional Knowledge Labels "*improve the quality of provenance, encourage communities to enrich records with their own traditional knowledge, and increase capacity for better understanding of equity and decision-making regarding re-use and circulation"* [120]. The provenance of any biocultural samples, collections, datasets, and traditional knowledge should be noted in full in metadata.

Although limited research has been conducted on access to agricultural omics benefits [113], we can learn from ethics frameworks for health and biomedical data, which can be adapted to the agricultural domain [121]. For example, Tiffin et al. [122] emphasize the need for data governance that protects vulnerable populations, especially in low-income and middle-income countries, when utilizing digital health data. Further, Mott et al. [101] discuss the use of homomorphic encryption for secure data sharing, which can facilitate the inclusion of private or sensitive data without compromising data confidentiality. This technology could be a key enabler in making data sharing more inclusive, especially when dealing with sensitive information from indigenous communities, as highlighted by Carroll et al. [120]

On the heels of many studies quantifying discrimination in academia [91], the big data community has a unique opportunity to build a field of research with fewer biases. Efforts should be directed towards creating centralized repositories that host diverse agricultural datasets, making it easier for researchers to locate and access relevant

information. Addressing issues related to data ownership and equitable access is vital if we are to reap all the benefits of data reuse as a global genomics community.

The future of data reuse is bright

Here, we have assessed challenges to reusing sequence-based agricultural datasets and presented possible future solutions regarding (meta)data availability, ownership, user resources, and equity. There is a growing demand for the reuse of published datasets and reinforcing the importance of well-structured databases to increase these numbers in the future. A change in global research culture that emphasizes the 'R' for reuse in FAIR would cause significant increases in data submissions, accompanied by more frequent reuse.

One of the biggest challenges of data reuse is to establish and enforce (meta)data standards and sharing requirements. Defined data standards and recommendations would address the issues of data quality, availability, sparsity of metadata, and formatting in the agricultural genomics field. The number of omics datasets is increasing every year and to keep the data well organized, following some standards can be helpful to enable reproducibility, with the added benefit of being good scientific practice. Other traditional knowledge management domains such as libraries, specifically data librarians may ultimately guide the creation of organizational standards. Maintaining these standards, as well as detailing important information that was cited throughout this article, may facilitate the reuse of omics data for future analysis. It may also aid in bringing all areas of agricultural research on equal footing when it comes to the benefits

of open science [123]. This will benefit future scientists and developers of applications and databases, contributing to science.

To aid in establishing best practices in the agricultural data field, we have compiled recommendations in a GitHub page [124], which we aim to keep updated with discussion points resulting from the AgBioData working group on data reuse. We invite any interested party to contribute to this community resource.

The focus of this (over)view of the status of data reuse in agricultural research has been sequence-based datasets. However, we acknowledge that many challenges and opportunities associated with these types of biological data are shared with non-sequence-based datasets. Indeed, these diverse data types come with their own unique set of challenges and rewards of reuse. Examples of these datasets include, and are not limited to, phenomes, metabolomes, proteomes, interactomes, enviromes, microbiomes, lipidomes, and glycomes. Additionally, many analyses include geographic, climate, and ecological data, which must also be considered for reuse purposes. Advances in artificial intelligence promise to allow for more knowledge to be gleaned from large, shared, interdisciplinary datasets. The omics revolution is still ongoing, and we must keep emerging data types in mind when considering reuse standards and platforms. It will be important to consider how such data types can be integrated with sequence-based data for future applications, further emphasizing the importance of complete metadata and biosample information currently deposited in databases. We, in the AgBioData DRWG, believe the future of data reuse is bright as

more datasets are reused successfully, contributing to the sustainability of agricultural research in the omics era.

Conclusions

Data reuse is beginning to yield exciting science across disciplines. Harnessing the power of large agricultural omics projects, like FarmGTEx [31] and Rice3K [32], has demonstrated the detailed knowledge that can be obtained from reuse. As many barriers to reuse keep falling, the biggest obstacle may continue to be the labor investment needed from the data producer (e.g., submitting data to repositories) and re-user (e.g., often convoluted process of obtaining data). Establishing more standards across data production, management, and sharing would pave the way to lowering the barrier of entry to the benefits of reuse. Many data producers are sharing their data, but there is a need for more incentives to encourage true FAIR compliance to facilitate reuse. Researcher skill level, one of the major barriers to reuse, needs to be bolstered with guidance and training programs, ensuring equity across all stakeholders in the global agricultural community. In addition, to ensure the maintenance of data availability, it is imperative that the scientific community continues to invest in data management infrastructure and resources. The future of data reuse will also benefit from the development of user-friendly tools and platforms that facilitate data discovery, access, and analysis.

The benefits are clear; data reuse facilitates the ability to ask big questions and provides community resources about genomes and phenomes that one group alone

cannot achieve. As more funding agencies are promoting data reuse, more scientists will see the exciting opportunities to solve grand challenges in biology. The next big breakthrough in predictive biology will likely require the integration of many diverse datasets. The future of data reuse in agriculture hinges on a collective commitment to data management, standards, infrastructure development, and collaboration between researchers. The open science principles are necessary to improve innovative research and sustainable agricultural practices. The data is out there to reuse; it is time to develop your innovative idea and run with the exciting datasets that are already available. The sky's the limit!

Declarations

Data availability

No additional analysis was conducted for this white paper. The recommendations resulting from analysis can be found on https://github.com/AgBioData/Data-Reuse.

Competing interests

The authors declare no competing interests.

Funding

We acknowledge funding from the USDA NIFA-AG2PI seed grant entitled “Harnessing Ag Genomics Data to link genotype to phenotype” as part of the USDA-NIFA awards 2020-70412-32615 and 2021-70412-35233, and to the AgBioData Consortium through the NSF for the Research Coordination Network project award abstract #2126334.

Acknowledgments

The authors wish to thank the AgBioData group for support and assistance in the logistics of the data reuse subgroup meetings. We also thank Dr. Mark Wilkinson (Industry Chair on Biotechnology, Polytechnic University of Madrid), Dr. Leonore Reiser (Principal Biocuration Scientist, Phoenix Bioinformatics) and Dr. Fiona McCarthy (Associate Professor, University of Arizona) for comments that greatly improved the manuscript. AH respectfully acknowledges the Erie, Haudenosaunee, Shawnee, Susquehannock, and Wahzhazhe Nations, as traditional caretakers of the lands she works on.

Authors' contributions

JEK initiated the collaboration, contributed to writing the manuscript, obtained funding for data reuse workshops, and chaired a working group to discuss data reuse needs and challenges. AH contributed to writing the manuscript, made the figures, and co-chaired the working group. CGE contributed to writing the manuscript. VLD contributed to writing the text for the interoperability section and revising the manuscript. PWH contributed to writing text for the metadata and ontologies section, and the future of data reuse section. BPu contributed to writing text for the sections benefits of data reuse, data availability, and future of data reuse. contributed to writing the text. TK contributed to writing text for "Towards interoperability via data formatting" and "Resource availability and user skill level". BPe contributed to the editing and revising the manuscript and future of data reuse section. EQR contributed to writing the equity and inclusion section. CD, DM, and CT contributed to editing and revising the manuscript.

References

1. Science Digital, Hahnel M, Smith G, Schoenenberger H, Scaplehorn N, Day L. The State of Open Data 2023. Digital Science; 2023 Nov.

2. McKiernan EC, Bourne PE, Brown CT, Buck S, Kenall A, Lin J, et al.. How open science helps researchers succeed. *eLife*. 2016; doi: 10.7554/eLife.16800.

3. Satam H, Joshi K, Mangrolia U, Waghoo S, Zaidi G, Rawool S, et al.. Next-Generation Sequencing Technology: Current Trends and Advancements. *Biology*. Multidisciplinary Digital Publishing Institute; 2023; doi: 10.3390/biology12070997.

4. Wu SZ, Al-Eryani G, Roden DL, Junankar S, Harvey K, Andersson A, et al.. A single-cell and spatially resolved atlas of human breast cancers. *Nat Genet*. Nature Publishing Group; 2021; doi: 10.1038/s41588-021-00911-1.

5. Sielemann K, Hafner A, Pucker B. The reuse of public datasets in the life sciences: potential risks and rewards. *PeerJ*. PeerJ Inc.; 2020; doi: 10.7717/peerj.9954.

6. Fernández-Ardèvol M, Rosales A. Quality Assessment and Biases in Reused Data. *American Behavioral Scientist*. 2022; doi: 10.1177/00027642221144855.

7. Devare M, Arnaud E, Antezana E, King B. Governing Agricultural Data: Challenges and Recommendations. In: Williamson HF, Leonelli S, editors. *Towards Responsible Plant Data Linkage: Data Challenges for Agricultural Research and Development*. Cham: Springer International Publishing;

8. Arita M, Karsch-Mizrachi I, Cochrane G. The international nucleotide sequence database collaboration. *Nucleic Acids Research*. 2021; doi: 10.1093/nar/gkaa967.

9. CNCB-NGDC Members and Partners. Database Resources of the National Genomics Data Center, China National Center for Bioinformation in 2024. *Nucleic Acids Research*. 2024; doi: 10.1093/nar/gkad1078.

10. Liu S, Gao Y, Canela-Xandri O, Wang S, Yu Y, Cai W, et al.. A multi-tissue atlas of regulatory variants in cattle. *Nature Genetics*. 2022; doi: 10.1038/s41588-022-01153-5.

11. Papoutsoglou EA, Faria D, Arend D, Arnaud E, Athanasiadis IN, Chaves I, et al.. Enabling reusability of plant phenomic datasets with MIAPPE 1.1. *New Phytol*. 2020; doi: 10.1111/nph.16544.

12. Hafner A, Mackenzie S. Re-analysis of publicly available methylomes using signal detection yields new information. *Sci Rep*. 2023; doi: 10.1038/s41598-023-30422-4.

13. Naithani S, Dikeman D, Garg P, Al-Bader N, Jaiswal P. Beyond gene ontology (GO): using biocuration approach to improve the gene nomenclature and functional annotation of rice S-domain kinase subfamily. *PeerJ*. 2021; doi: 10.7717/peerj.11052.

14. Rempel A, Choudhary N, Pucker B. KIPEs3: Automatic annotation of biosynthesis pathways. Ezura H, editor. *PLoS ONE*. 2023; doi: 10.1371/journal.pone.0294342.

15. He Z, Luo Y, Zhou X, Zhu T, Lan Y, Chen D. scPlantDB: a comprehensive database for exploring cell types and markers of plant cell atlases. *Nucleic Acids Research*. 2024; doi: 10.1093/nar/gkad706.

16. Chen Y, Zhang X, Peng X, Jin Y, Ding P, Xiao J, et al.. SPEED: Single-cell Pan-species atlas in the light of Ecology and Evolution for Development and Diseases. *Nucleic Acids Research*. 2023; doi: 10.1093/nar/gkac930.

17. Fu L-Y, Zhu T, Zhou X, Yu R, He Z, Zhang P, et al.. ChIP-Hub provides an integrative platform for exploring plant regulome. *Nat Commun*. 2022; doi: 10.1038/s41467-022-30770-1.

18. Tenopir C, Rice NM, Allard S, Baird L, Borycz J, Christian L, et al.. Data sharing, management, use, and reuse: Practices and perceptions of scientists worldwide. Lozano S, editor. *PLoS ONE*. 2020; doi: 10.1371/journal.pone.0229003.

19. Gomes DGE, Pottier P, Crystal-Ornelas R, Hudgins EJ, Foroughirad V, Sánchez-Reyes LL, et al.. Why don’t we share data and code? Perceived barriers and benefits to public archiving practices. 2022;

20. LaFlamme M, Poetz M, Spichtinger D. Seeing oneself as a data reuser: How subjectification activates the drivers of data reuse in science. Fàbregues S, editor. *PLoS ONE*. 2022; doi: 10.1371/journal.pone.0272153.

21. Senft M, Stahl U, Svoboda N. Research data management in agricultural sciences in Germany: We are not yet where we want to be. Pulvento C, editor. *PLoS ONE*. 2022; doi: 10.1371/journal.pone.0274677.

22. Verhulst S, Young A. Identifying and addressing data asymmetries so as to enable (better) science. *Front Big Data*. 2022; doi: 10.3389/fdata.2022.888384.

23. Wilkinson MD, Dumontier M, Aalbersberg IjJ, Appleton G, Axton M, Baak A, et al.. The FAIR Guiding Principles for scientific data management and stewardship. *Sci Data*. 2016; doi: 10.1038/sdata.2016.18.

24. . Announcement: Where are the data? *Nature*. 2016; doi: 10.1038/537138a.

25. . Open Data in a Big Data World. *Chemistry International*. 2016; doi: doi:10.1515/ci-2016-0208.

26. CODATA, Hodson, Simon, Mons, Barend, Uhlir, Paul, Zhang, Lili.. The Beijing Declaration on Research Data.

27. Nosek BA, Alter G, Banks GC, Borsboom D, Bowman SD, Breckler SJ, et al.. Promoting an open research culture. *Science*. 2015; doi: 10.1126/science.aab2374.

28. OECD. Enhanced Access to Publicly Funded Data for Science, Technology and Innovation. OECD;

29. Lewin HA, Richards S, Lieberman Aiden E, Allende ML, Archibald JM, Bálint M, et al.. The Earth BioGenome Project 2020: Starting the clock. *Proceedings of the National Academy of Sciences*. Proceedings of the National Academy of Sciences; 2022; doi: 10.1073/pnas.2115635118.

30. : Vertebrate Genomes Project. Nature. https://www.nature.com/collections/cabiagjdfj (2021). Accessed 2023 Dec 21.

31. . The CattleGTEx atlas reveals regulatory mechanisms underlying complex traits. *Nature Genetics*. 2022; doi: 10.1038/s41588-022-01155-3.

32. Day A, Poplin R. Analyzing 3024 rice genomes characterized by DeepVariant. Google Cloud Blog.

33. Rodrigo A, Alberts S, Cranston K, Kingsolver J, Lapp H, McClain C, et al.. Science Incubators: Synthesis Centers and Their Role in the Research Ecosystem. *PLOS Biology*. Public Library of Science; 2013; doi: 10.1371/journal.pbio.1001468.

34. Rexroad C, Vallet J, Matukumalli LK, Reecy J, Bickhart D, Blackburn H, et al.. Genome to Phenome: Improving Animal Health, Production, and Well-Being – A New USDA Blueprint for Animal Genome Research 2018–2027. *Frontiers in Genetics*. 102019;

35. Tuggle CK, Clarke JL, Murdoch BM, Lyons E, Scott NM, Beneš B, et al.. Current challenges and future of agricultural genomes to phenomes in the USA. *Genome Biol*. 2024; doi: 10.1186/s13059-023-03155-w.

36. Tuggle CK, Clarke J, Dekkers JCM, Ertl D, Lawrence-Dill CJ, Lyons E, et al.. The Agricultural Genome to Phenome Initiative (AG2PI): creating a shared vision across crop and livestock research communities. *Genome Biology*. 2022; doi: 10.1186/s13059-021-02570-1.

37. : AG2PI. https://www.ag2pi.org Accessed 2024 Apr 1.

38. : Understanding the Rules of Life. https://www.nsf.gov/news/special_reports/big_ideas/life.jsp Accessed 2024 Apr 1.

39. Chen L, Qiu Q, Jiang Y, Wang K, Lin Z, Li Z, et al.. Large-scale ruminant genome sequencing provides insights into their evolution and distinct traits. *Science*. American Association for the Advancement of Science; 2019; doi: 10.1126/science.aav6202.

40. Leebens-Mack JH, Barker MS, Carpenter EJ, Deyholos MK, Gitzendanner MA, Graham SW, et al.. One thousand plant transcriptomes and the phylogenomics of green plants. *Nature*. Nature Publishing Group; 2019; doi: 10.1038/s41586-019-1693-2.

41. Zhang G. Bird sequencing project takes off. *Nature*. Nature Publishing Group; 2015; doi: 10.1038/522034d.

42. Ed Kalbfleisch, Stephanie McKay, Brenda Murdoch, David L. Adelson, Diego Almansa, Gabrielle Becker, et al.. RT2T: A Global Collaborative Project to Study Chromosomal Evolution in the Suborder Ruminantia. *Research Square*. 2024; doi: https://doi.org/10.21203/rs.3.rs-3918604/v2.

43. : AgBioData. https://www.agbiodata.org/ Accessed 2024 Apr 1.

44. Deng CH, Naithani S, Kumari S, Cobo-Simón I, Quezada-Rodríguez EH, Skrabisova M, et al.. Genotype and phenotype data standardization, utilization and integration in the big data era for agricultural sciences. *Database*. 2023; doi: 10.1093/database/baad088.

45. Harper L, Campbell J, Cannon EKS, Jung S, Poelchau M, Walls R, et al.. AgBioData consortium recommendations for sustainable genomics and genetics databases for agriculture. *Database (Oxford)*. 2018; doi: 10.1093/database/bay088.

46. Saha S, Cain S, Cannon EKS, Dunn N, Farmer A, Hu Z-L, et al.. Recommendations for extending the GFF3 specification for improved interoperability of genomic data. *arXiv*. 2022; doi: arXiv:2202.07782.

47. Moorhead JE, Rao PV, Anusavice KJ. Guidelines for experimental studies. *Dental Materials*. 1994; doi: 10.1016/0109-5641(94)90021-3.

48. Delgado A. An economy of details: standards and data reusability. *Synthetic Biology*. 2023; doi: 10.1093/synbio/ysac030.

49. Curty RG, Crowston K, Specht A, Grant BW, Dalton ED. Attitudes and norms affecting scientists' data reuse. *PLOS ONE*. Public Library of Science; 2017; doi: 10.1371/journal.pone.0189288.

50. : Genomic Data Commons. https://gdc.cancer.gov/ Accessed 2024 Apr 1.

51. : MIAME. https://www.ncbi.nlm.nih.gov/geo/info/MIAME.html Accessed 2024 Apr 1.

52. Schurch NJ, Schofield P, Gierliński M, Cole C, Sherstnev A, Singh V, et al.. How many biological replicates are needed in an RNA-seq experiment and which differential expression tool should you use? *RNA*. 2016; doi: 10.1261/rna.053959.115.

53. Schuurman N, Leszczynski A. Ontologies for Bioinformatics. *Bioinform Biol Insights*. 2:187–2002008;

54. Clarke JL, Cooper LD, Poelchau MF, Berardini TZ, Elser J, Farmer AD, et al.. Data sharing and ontology use among agricultural genetics, genomics, and breeding

databases and resources of the Agbiodata Consortium. *Database*. 2023; doi: 10.1093/database/baad076.

55. : FAANG Ontology Improver. https://data.faang.org/ontology Accessed 2024 Apr 1.

56. : INSDC. https://www.ncbi.nlm.nih.gov/biosample/docs/attributes/ Accessed 2024 Apr 1.

57. Brunak S, Danchin A, Hattori M, Nakamura H, Shinozaki K, Matise T, et al.. Nucleotide Sequence Database Policies. *Science*. American Association for the Advancement of Science; 2002; doi: 10.1126/science.298.5597.1333b.

58. Deckard J, McDonald CJ, Vreeman DJ. Supporting interoperability of genetic data with LOINC. *Journal of the American Medical Informatics Association*. 2015; doi: 10.1093/jamia/ocu012.

59. Ćwiek-Kupczyńska H, Altmann T, Arend D, Arnaud E, Chen D, Cornut G, et al.. Measures for interoperability of phenotypic data: minimum information requirements and formatting. *Plant Methods*. 2016; doi: 10.1186/s13007-016-0144-4.

60. Jenkins GB, Beckerman AP, Bellard C, Benítez-López A, Ellison AM, Foote CG, et al.. Reproducibility in ecology and evolution: Minimum standards for data and code. *Ecology and Evolution*. John Wiley & Sons, Ltd; 2023; doi: 10.1002/ece3.9961.

61. Wang Y, Sarfraz I, Teh WK, Sokolov A, Herb BR, Creasy HH, et al.. Matrix and analysis metadata standards (MAMS) to facilitate harmonization and reproducibility of single-cell data.

62. Cernava T, Rybakova D, Buscot F, Clavel T, McHardy AC, Meyer F, et al.. Metadata harmonization–Standards are the key for a better usage of omics data for integrative microbiome analysis. *Environmental Microbiome*. 2022; doi: 10.1186/s40793-022-00425-1.

63. : nfdi4plants. https://www.nfdi4plants.de) Accessed 2024 Apr 1.

64. O'Leary NA, Cox E, Holmes JB, Anderson WR, Falk R, Hem V, et al.. Exploring and retrieving sequence and metadata for species across the tree of life with NCBI Datasets. *Scientific Data*. 2024; doi: 10.1038/s41597-024-03571-y.

65. Zhang H. Overview of Sequence Data Formats. In: Mathé E, Davis S, editors. *Statistical Genomics*. New York, NY: Springer New York;

66. Li H, Handsaker B, Wysoker A, Fennell T, Ruan J, Homer N, et al.. The Sequence Alignment/Map format and SAMtools. *Bioinformatics*. 2009; doi: 10.1093/bioinformatics/btp352.

67. : SAMv1. https://samtools.github.io/hts-specs/SAMv1.pdf) Accessed 2024 Apr 1.

68. Beier S, Fiebig A, Pommier C, Liyanage I, Lange M, Kersey P, et al.. Recommendations for the formatting of Variant Call Format (VCF) files to make plant genotyping data FAIR [version 2; peer review: 2 approved]. *F1000Research*. 2022; doi: 10.12688/f1000research.109080.2.

69. Danecek P, Auton A, Abecasis G, Albers CA, Banks E, DePristo MA, et al.. The variant call format and VCFtools. *Bioinformatics*. 2011; doi: 10.1093/bioinformatics/btr330.

70. : GTF. http://mblab.wustl.edu/GTF22.html Accessed 2024 Apr 1.

71. : GFF3. https://github.com/The-Sequence-Ontology/Specifications/blob/master/gff3.md Accessed 2024 Apr 1.

72. : BED. https://genome.ucsc.edu/FAQ/FAQformat.html#format1 Accessed 2024 Apr 1.

73. : Ensembl GFF. http://useast.ensembl.org/info/website/upload/gff.html Accessed 2024 Apr 1.

74. : Genome Analysis Toolkit. https://gatk.broadinstitute.org/hc/en-us

75. Lee S-G, Na D, Park C. Comparability of reference-based and reference-free transcriptome analysis approaches at the gene expression level. *BMC Bioinformatics*. 2021; doi: 10.1186/s12859-021-04226-0.

76. Parra-Salazar A, Gomez J, Lozano-Arce D, Reyes-Herrera PH, Duitama J. Robust and efficient software for reference-free genomic diversity analysis of genotyping-by-sequencing data on diploid and polyploid species. *Molecular Ecology Resources*. 2022; doi: 10.1111/1755-0998.13477.

77. Petri AJ, Sahlin K. isONform: reference-free transcriptome reconstruction from Oxford Nanopore data. *Bioinformatics*. 2023; doi: 10.1093/bioinformatics/btad264.

78. Afzal M, Sielaff M, Distler U, Schuppan D, Tenzer S, Longin CFH. Reference proteomes of five wheat species as starting point for future design of cultivars with lower allergenic potential. *npj Science of Food*. 2023; doi: 10.1038/s41538-023-00188-0.

79. Bar N, Korem T, Weissbrod O, Zeevi D, Rothschild D, Leviatan S, et al.. A reference map of potential determinants for the human serum metabolome. *Nature*. 2020; doi: 10.1038/s41586-020-2896-2.

80. Ambroise J, Irenge LM, Durant J-F, Bearzatto B, Bwire G, Stine OC, et al.. Backward compatibility of whole genome sequencing data with MLVA typing using a new MLVAtype shiny application for Vibrio cholerae. *PLOS ONE*. Public Library of Science; 2019; doi: 10.1371/journal.pone.0225848.

81. Bletz S, Mellmann A, Rothgänger J, Harmsen D. Ensuring backwards compatibility: traditional genotyping efforts in the era of whole genome sequencing. *Clinical Microbiology and Infection*. 2015; doi: 10.1016/j.cmi.2014.11.005.

82. Gordon M, Yakunin E, Valinsky L, Chalifa-Caspi V, Moran-Gilad J. A bioinformatics tool for ensuring the backwards compatibility of Legionella pneumophila typing in the genomic era. *Clinical Microbiology and Infection*. 2017; doi: 10.1016/j.cmi.2017.01.002.

83. : Protocols.io. https://www.protocols.io Accessed 2024 Apr 1.

84. de Farias TM, Wollbrett J, Robinson-Rechavi M, Bastian F. Lessons learned to boost a bioinformatics knowledge base reusability, the Bgee experience. *arXiv*. 2023; doi: arXiv.2303.12329.

85. Tedersoo L, Küngas R, Oras E, Köster K, Eenmaa H, Leijen Ä, et al.. Data sharing practices and data availability upon request differ across scientific disciplines. *Sci Data*. Nature Publishing Group; 2021; doi: 10.1038/s41597-021-00981-0.

86. Eckert EM, Di Cesare A, Fontaneto D, Berendonk TU, Bürgmann H, Cytryn E, et al.. Every fifth published metagenome is not available to science. *PLoS Biol*. 2020; doi: 10.1371/journal.pbio.3000698.

87. Stodden V, Seiler J, Ma Z. An empirical analysis of journal policy effectiveness for computational reproducibility. *Proc Natl Acad Sci U S A*. 2018; doi: 10.1073/pnas.1708290115.

88. Ahmed M, Kim HJ, Kim DR. Maximizing the utility of public data. *Front Genet*. 2023; doi: 10.3389/fgene.2023.1106631.

89. Koppad S, B A, Gkoutos GV, Acharjee A. Cloud Computing Enabled Big Multi-Omics Data Analytics. *Bioinform Biol Insights*. SAGE Publications Ltd STM; 2021; doi: 10.1177/11779322211035921.

90. : Global Biodata Coalition. https://globalbiodata.org/ Accessed 2024 Apr 1.

91. Harper L, Campbell J, Cannon EKS, Jung S, Poelchau M, Walls R, et al.. AgBioData consortium recommendations for sustainable genomics and genetics databases for agriculture. *Database*. 2018; doi: 10.1093/database/bay088.

92. Groth P, Cousijn H, Clark T, Goble C. FAIR Data Reuse – the Path through Data Citation. *Data Intellegence*. 2020; doi: 10.1162/dint_a_00030.

93. : Open Science Framework. https://osf.io Accessed 2024 Apr 1.

94. : DataCite. http://corpus.datacite.org/). Accessed 2024 Apr 1.

95. Wood-Charlson EM, Crockett Z, Erdmann C, Arkin AP, Robinson CB. Ten simple rules for getting and giving credit for data. Schwartz R, editor. *PLoS Comput Biol*. 2022; doi: 10.1371/journal.pcbi.1010476.

96. Perez-Riverol Y, Zorin A, Dass G, Vu M-T, Xu P, Glont M, et al.. Quantifying the impact of public omics data. *Nature Communications*. 2019; doi: 10.1038/s41467-019-11461-w.

97. Ray KS, Zurn P, Dworkin JD, Bassett DS, Resnik DB. Citation bias, diversity, and ethics. *Accountability in Research*. Taylor & Francis; 2022; doi: 10.1080/08989621.2022.2111257.

98. Zimmerman S: Corteva lawsuit accuses gene-editing startup of stealing seeds. Agriculture Dive. https://www.agriculturedive.com/news/corteva-lawsuit-inari-steal-seeds-gene-editing/695605/ (2023). Accessed 2023 Dec 21.

99. Blatt M, Gusev A, Polyakov Y, Goldwasser S. Secure large-scale genome-wide association studies using homomorphic encryption. *Proc Natl Acad Sci U S A*. 2020; doi: 10.1073/pnas.1918257117.

100. Konečný J, McMahan B, Ramage D. Federated Optimization:Distributed Optimization Beyond the Datacenter. *arXiv*. 2015; doi: arXiv:1511.03575.

101. Mott R, Fischer C, Prins P, Davies RW. Private Genomes and Public SNPs: Homomorphic Encryption of Genotypes and Phenotypes for Shared Quantitative Genetics. *Genetics*. 2020; doi: 10.1534/genetics.120.303153.

102. Zhao T, Wang F, Mott R, Dekkers J, Cheng H. Using encrypted genotypes and phenotypes for collaborative genomic analyses to maintain data confidentiality. *Genetics*. 2023; doi: 10.1093/genetics/iyad210.

103. : White House Office of Science and Technology Policy Issues Guidance to Make Federally Funded Research Freely Available Without Delay. https://www.whitehouse.gov/ostp/news-updates/2022/08/25/ostp-issues-guidance-to-make-federally-funded-research-freely-available-without-delay/ (2022). Accessed 2024 Apr 1.

104. Smyth SJ, Macall DM, Phillips PWB, de Beer J. Implications of biological information digitization: Access and benefit sharing of plant genetic resources. *The Journal of World Intellectual Property*. John Wiley & Sons, Ltd; 2020; doi: 10.1111/jwip.12151.

105. Wynberg R, Andersen R, Laird S, Kusena K, Prip C, Westengen OT. Farmers' Rights and Digital Sequence Information: Crisis or Opportunity to Reclaim Stewardship Over Agrobiodiversity? *Frontiers in Plant Science*. 122021;

106. : The Nagoya Protocol. https://www.cbd.int/abs/ Accessed 2024 Apr 1.

107. : International Treaty on Plant Genetic Resources for Food and Agriculture. https://www.fao.org/plant-treaty/en/ Accessed 2024 Apr 1.

108. : African Biogenome Project. https://africanbiogenome.org/ Accessed 2024 Apr 1.

109. Wolff K, Friedhoff R, Schwarzer F, Pucker B. Data literacy in genome research. *Journal of Integrative Bioinformatics*. 2023; doi: 10.1515/jib-2023-0033.

110. : DataWorks! https://www.herox.com/dataworks Accessed 2024 Apr 1.

111. : Research Parasite. https://researchparasite.com/ Accessed 2024 Apr 1.

112. Weersink A, Fraser E, Pannell D, Duncan E, Rotz S. Opportunities and Challenges for Big Data in Agricultural and Environmental Analysis. *Annual Review of Resource Economics*. 2018; doi: 10.1146/annurev-resource-100516-053654.

113. Harris J, Tan W, Mitchell B, Zayed D. Equity in agriculture-nutrition-health research: a scoping review. *Nutrition Reviews*. 2022; doi: 10.1093/nutrit/nuab001.

114. Friendly M, Denis DJ: Milestones in the history of thematic cartography, statistical graphics, and data visualization. http://www.datavis.ca/milestones/ (2001). Accessed 2023 Dec 21.

115. Li Q. Embodying Data: Chinese Aesthetics, Interactive Visualization and Gaming Technologies. Singapore: Springer;

116. Pasquetto IV, Borgman CL, Wofford MF. Uses and Reuses of Scientific Data: The Data Creators' Advantage. *Harvard Data Science Review*. 2019; doi: 10.1162/99608f92.fc14bf2d.

117. Hofstra B, Kulkarni VV, Munoz-Najar Galvez S, He B, Jurafsky D, McFarland DA. The Diversity–Innovation Paradox in Science. *Proceedings of the National Academy of*

Sciences. Proceedings of the National Academy of Sciences; 2020; doi: 10.1073/pnas.1915378117.

118. Carroll SR, Garba I, Figueroa-Rodríguez OL, Holbrook J, Lovett R, Materechera S, et al.. The CARE Principles for Indigenous Data Governance. *Data Science Journal*. 2020; doi: 10.5334/dsj-2020-043.

119. : Local Contexts. https://localcontexts.org Accessed 2024 Apr 1.

120. Carroll SR, Herczog E, Hudson M, Russell K, Stall S. Operationalizing the CARE and FAIR Principles for Indigenous data futures. *Scientific Data*. 2021; doi: 10.1038/s41597-021-00892-0.

121. Xafis V, Schaefer GO, Labude MK, Brassington I, Ballantyne A, Lim HY, et al.. An Ethics Framework for Big Data in Health and Research. *Asian Bioeth Rev*. 2019; doi: 10.1007/s41649-019-00099-x.

122. Tiffin N, George A, LeFevre AE. How to use relevant data for maximal benefit with minimal risk: digital health data governance to protect vulnerable populations in low-income and middle-income countries. *BMJ Global Health*. BMJ Specialist Journals; 2019; doi: 10.1136/bmjgh-2019-001395.

123. Muñoz-Tamayo R, Nielsen BL, Gagaoua M, Gondret F, Krause ET, Morgavi DP, et al.. Seven steps to enhance Open Science practices in animal science. Nelson KE, editor. *PNAS Nexus*. 2022; doi: 10.1093/pnasnexus/pgac106.

124. AgBioData: AgBioData Data Reuse Working Group’s Reccommendations. https://github.com/AgBioData/Data-Reuse Accessed 2024 Apr 1.

Fig. 1

Click here to access/download;Figure;Fig1.png

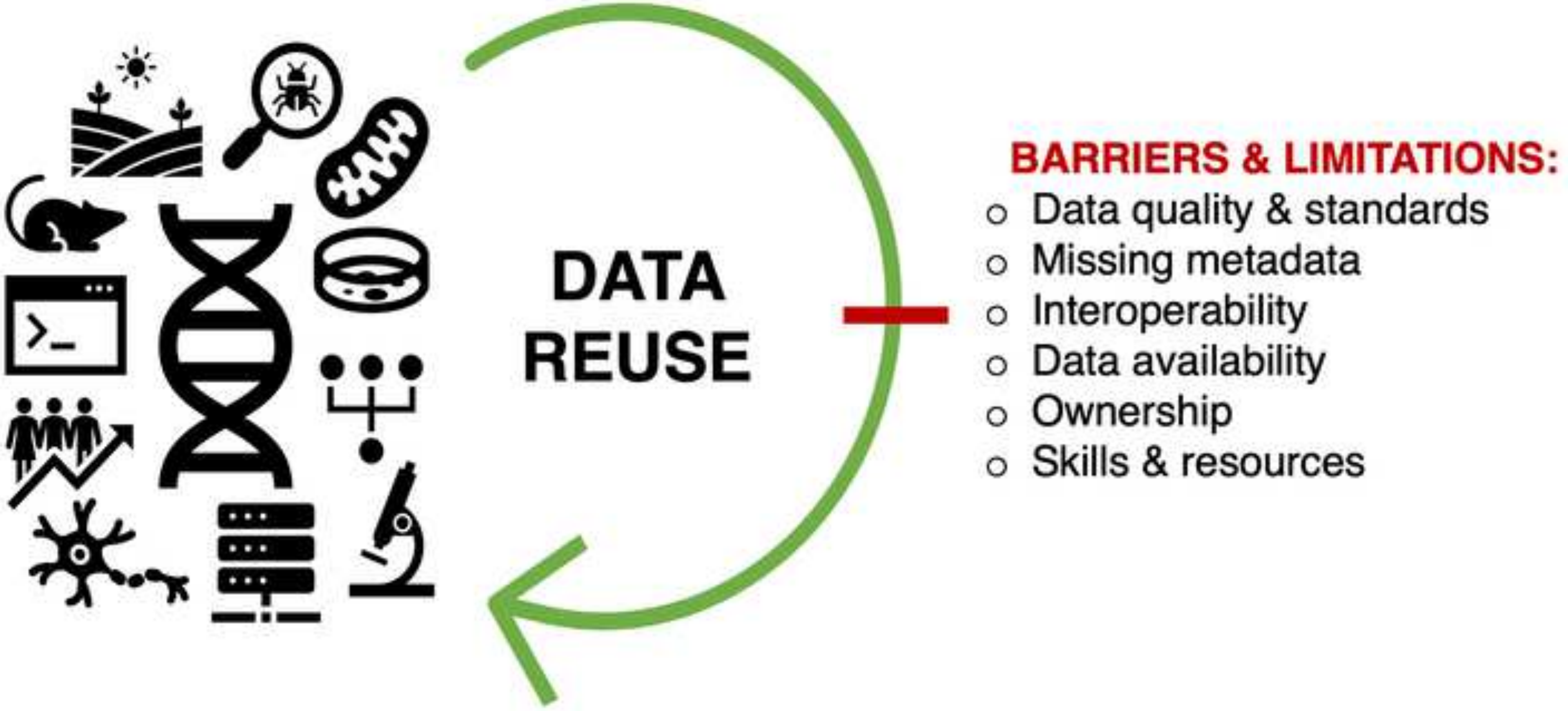

Fig. 2

Click here to access/download;Figure;Fig2.png

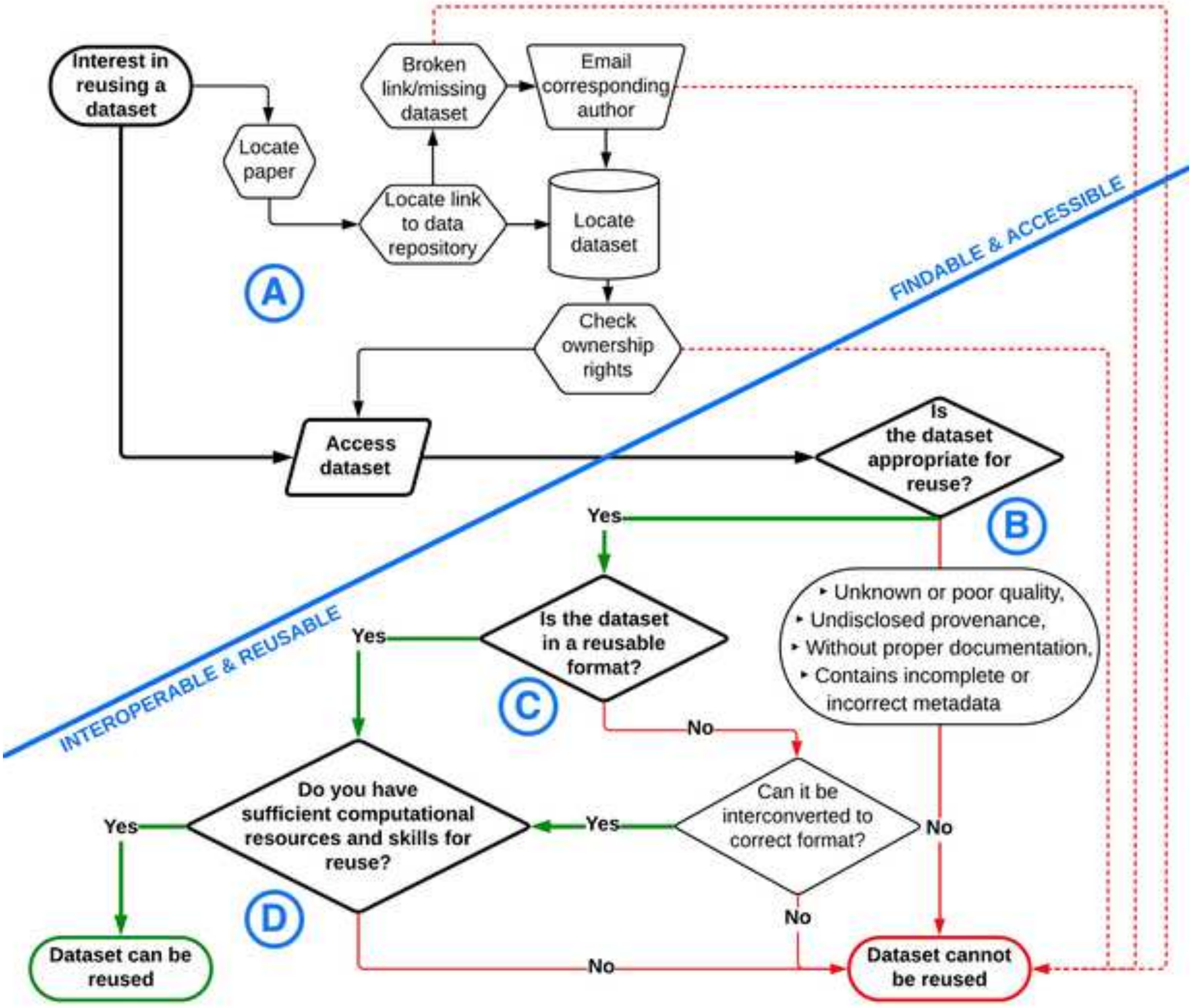

Fig. 3 Click here to access/download;Figure;Fig3.png

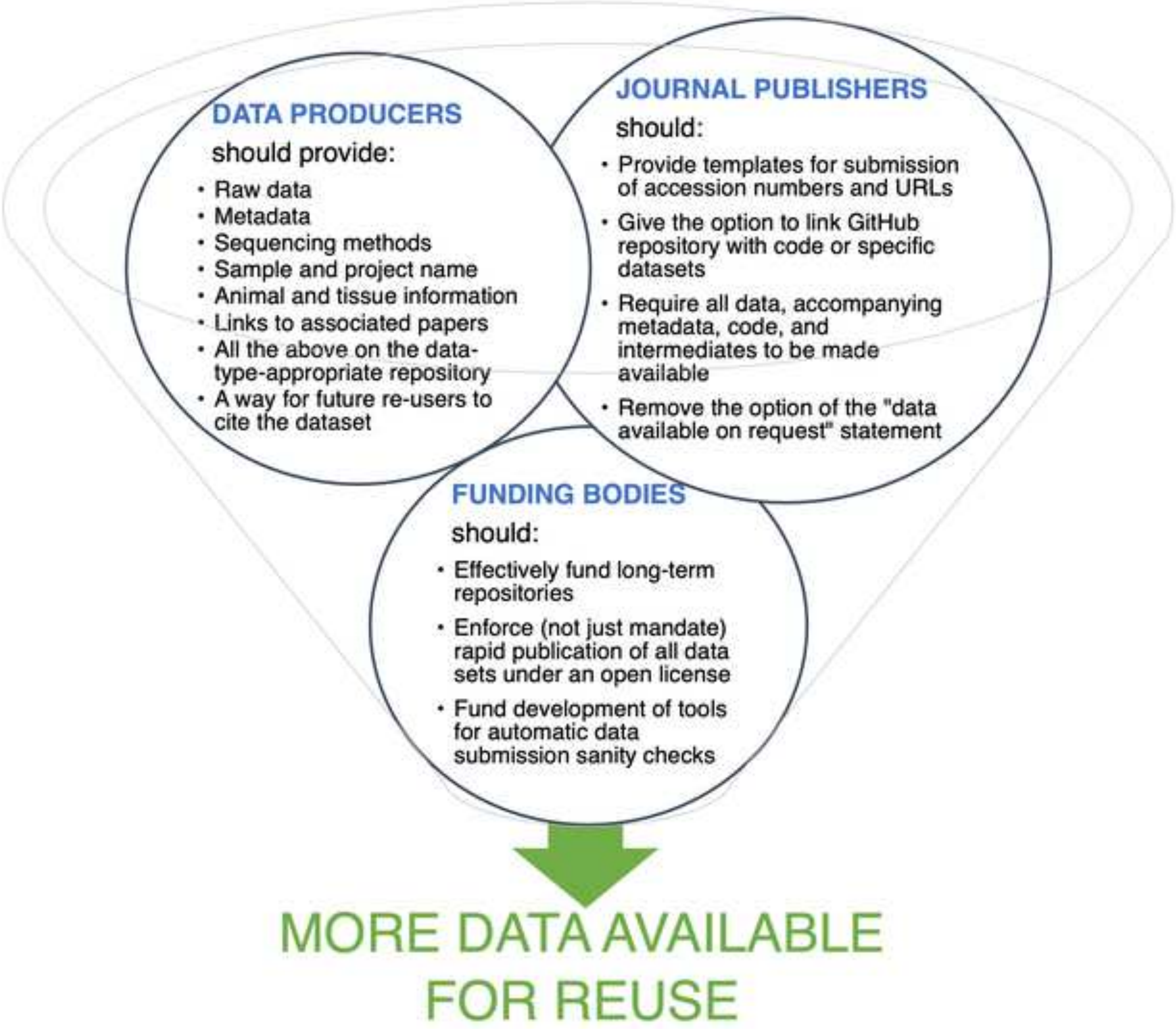

Fig. 4

Click here to access/download;Figure;Fig4.png

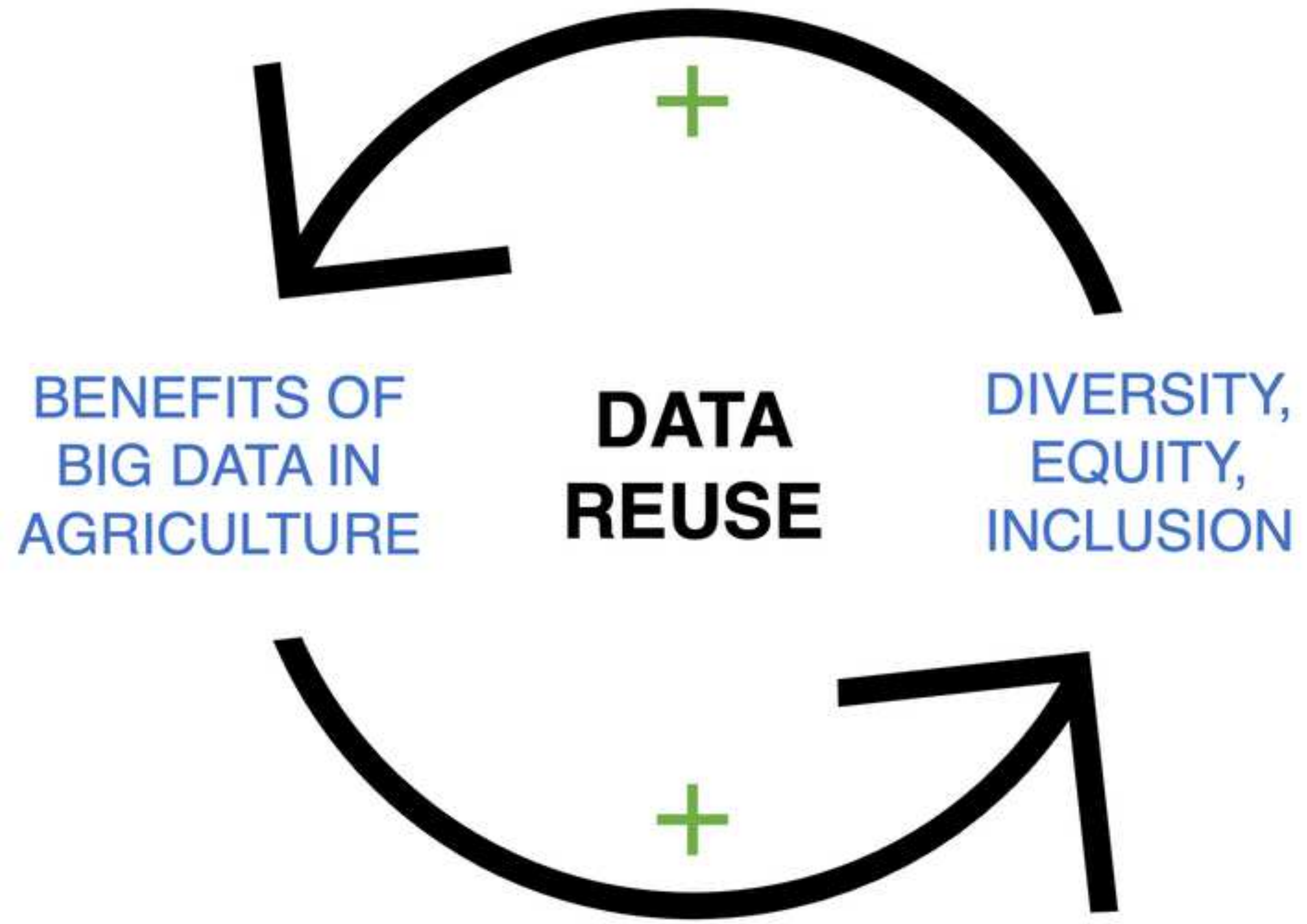

Supplement: giae106_GIGA-D-24-00228_Revision_1 [file giae106_giga-d-24-00228_revision_1.pdf]
