# Supplementary material for: Data reuse in agricultural genomics research: challenges and recommendations
Source: Gigascience. 2025 Jan 13;14:giae106. doi: 10.1093/gigascience/giae106 (PMC11727710; doi:10.1093/gigascience/giae106)
Supplement: giae106_Response_to_Reviewer_Comments_Original_Submission [file giae106_response_to_reviewer_comments_original_submission.pdf]

Author's Response To Reviewer Comments

Dear Reviewers,
Thank you for your efforts to review and provide suggestions to improve our manuscript. Please find point by point comments below for each suggest edit.

Best regards,
James Koltes and Alenka Haefner (for the authors)

Reviewer #1: The manuscript titled "Data reuse in agricultural genomics research: challenges and recommendations" provides an in-depth and timely analysis of the key challenges associated with data reuse in agricultural genomics, offering a set of actionable recommendations to improve these practices. The authors successfully identify and discuss critical obstacles such as data standards, metadata deficiencies, data interoperability, and equity issues, contributing valuable insights to the ongoing discourse in this area. While the manuscript is well-structured and presents a thorough examination of the subject, there are several areas that require further attention:

Major Points:

1. Lines 53-57: The manuscript references several major genomic data centers, but it omits the National Genomics Data Center (NGDC, https://ngdc.cncb.ac.cn/) and the Genome Sequence Archive (GSA, https://ngdc.cncb.ac.cn/gsa/). These should be included to provide a more comprehensive overview of available resources.

Thank you for your comment, we have now added the importance China National Center for Bioinformation's resources, including NGDc and GSA, to the international community.

2. Line 65: The examples of data reuse provided in the manuscript are helpful, but additional references would further strengthen this section. Consider including more examples from plant genomics, such as those found in these studies: 10.1093/nar/gkad706, 10.1093/nar/gkac930, and 10.1038/s41467-022-30770-1. The authors could explore more ...

Thank you for the additional examples of successful reuse. We have added plant examples that are agriculturally significant, including the references you provide. While two paragraphs in the Background section focus on these benefits, the main aim of our paper is not to argue the benefits of reuse as these are well-known and established in the field. That is why we refrain from expounding upon individual examples due previous reviews described in-depth the benefits of reuse (Sielemann et al., 2021).

3. While the manuscript covers a broad spectrum of challenges, certain sections would benefit from more in-depth analysis. The discussion on equity issues, for instance, is somewhat superficial. Expanding this section with detailed examples or case studies would provide a more robust understanding of the equity-related challenges in data reuse.

As we point out, the research on equity in big data has been very limited to date, particularly in the agricultural field. We have mentioned several studies addressing the issue (Harris et al., Xafis et al., Tiffin et al., Mott et al., Carroll et al.) and provided examples of how access issues can be addressed throughout this section. We find that the paragraphs on the issue of governance of Indigenous data is a very pertinent example and one applicable to many reuse cases a reader might encounter in agricultural genomics research.

4. The manuscript would be significantly strengthened by incorporating specific case studies or examples where data reuse has been successfully implemented in agricultural genomics. These practical illustrations would enhance the manuscript's applicability and offer readers tangible insights into the implementation of the recommendations.

Thank you for this constructive comment. While we have mentioned several examples of successful data reuse, we now expounded upon these in-text (lines 114-118 and 128-141). Please also see our response to comment no. 2.

5. The introduction effectively sets the stage by underscoring the importance of data reuse in the context of open science. However, it would benefit from a brief historical overview of the evolution of data reuse practices, especially within the agricultural genomics domain. This context would help readers appreciate the current state of data reuse and the progress made thus far.

Thank you for this suggestion. We have edited the section on the history of data reuse practices to better describe how it pertains to agricultural genomics (lines 17-22) and direct the reader to an in-depth overview by Sielemann et al. (2021).

6. The discussion on metadata deficiencies is well-crafted, but it could be further enriched by addressing recent advancements in metadata standards. Discussing how these innovations could mitigate current challenges would provide a more balanced and forward-looking perspective.

Thank you, we have now added more examples of advancements in metadata standards in other big data communities (line 312), which provides an optimistic outlook for the agricultural community. We also highlight the new NCBI Datasets initiative (lines 328-332).

7. The recommendations offered are thorough and relevant, yet the manuscript would be more practical if it distinguished between short-term and long-term solutions. This distinction would help readers and stakeholders prioritize actions based on their available resources and infrastructure.

Thank you for your assessment of our recommendations for solutions. We have added several qualifiers throughout the manuscript to distinguish between short- and long-term solutions (lines 358, 366, 480, 503).

Reviewer #2: In this manuscript, the authors explored the challenges and recommendations regarding data reuse. It identifies limitations related to data standards, metadata deficiencies, data interoperability, data ownership, data availability, user skill levels, resource availability, and equity issues, and suggests solutions that relevant stakeholders can implement. Additionally, the article offers an optimistic outlook on the future of data reuse in genomics and transcriptomics.

Although the authors had concluded the current problems during the data usage, but the most important things were solved to these problems, they didn't give any solutions.

Thank you for your comment. However, we disagree that we did not provide any solutions to the problems we describe. The paper is structured based on providing many viable solutions to each of the problems we describe. These were also recognized by reviewer #1. As you mention, our aim was to provide and optimistic outlook which would not have been possible without providing practical recommendations for the community to solve the problems we identified. Each main-body section describing individual barriers to data reuse (Fig. 1) ends in a paragraph describing solutions to the barrier. Not all solutions apply to the data reuser – many have to be implemented by other stakeholders, e.g. publishers, which we have pointed out in the relevant sections.

Currently, we have different type of data, eg. RNA-data, Protein data, Metabolic data, Stero-seq data, with different type of data, we need individual solutions for each type.

We completely agree with the need to have data formatting solutions tailored for different data types. As sequence-based data is so common across so many fields and additional work is done to share this data, it makes sense to focus on these data where the biggest impact can be made, before tackling the more diverse and stratified data types. We agree that re-use of these other data types is important but will require additional considerations.

As elaborated in the Background, our paper focuses on genomic sequence data due to its ubiquity and

importance to the agricultural community. Specifically, the manuscript states:

“While this perspective focuses on sequence- based data, it is important to acknowledge the issues facing phenotypic data reuse, particularly the prevalent ad hoc formats, lack of archives for storing and accessing data, and inability to share phenotype and genotype data together (due to agreements with industry or lack of infrastructure).”

The AgBioData initiative’s data reuse and other working groups are actively working on issues of other data types. While we mention other data types where pertinent, expounded in Future of data reuse is bright section, we consider in-depth analysis of them beyond the scope of this paper.

In the part of data formatting, the authors didn't clarify the other types of data not only for the normal genome data. I think they should add this
part.

We are unclear on what you are referencing here and assume you are referring to the section entitled “Towards interoperability via data formatting”. Our response to the previous comment would also apply here - this review is focusing on sequence-based technology data reuse which still requires considerable forethought to improve data reuse.

In the manuscript, the author said that the process files need also should be upload, I think this can't be realistic. Researchers can upload the data, as well as with some script, otherwise the database need a large storage.

We believe you are is commenting on the passage below (lines 470 – 481). We believe this section honors and raises similar concerns as those expressed by the reviewer.

“Being able to demonstrate reproducibility in analysis is important, and too often proves impossible [87], but it is equally important that the datasets achieve their full utility potential through reuse for novel purposes [88]. The processes that are performed to analyze the raw data are often beyond the computational resources and skills available to most researchers who could benefit from them. Therefore, it may be useful to make processed data, such as transcriptome and genome sequence assemblies, genomic variants, and peaks identified using technologies like chromatin immunoprecipitation sequencing (ChIP-seq), available along with the underlying reads whenever possible. However, storing intermediates and final products of pipelines comes at the cost of increasing the amount of necessary disk space, an important trade-off to consider. A possible short-term solution to this bottleneck to reuse is to make all code used in the computational analysis available alongside raw and/or processed datasets.”

The intent in this portion of the manuscript was not to imply that all processed data should be made public and it is mentioned specifically in this passage that the computational demands would be too high to not prioritize what processed data should be shared.

However, our team argues that if intermediate processed files are important to the overall results or interpretation of the study, they should not only be made public, but be made machine readable and reusable (i.e., interoperable). For example, if a high-quality transcriptomes, catalogue of genome variants, or epigenetic variants are identified in a study, these results would serve as important resources to the scientific community. Thus, they would be valuable for reuse as support for new studies, or foundations for new research.

We believe that under FAIR standards, researchers and databases should strive for complete accessibility and reusability. That requires that, as you point out, at minimum all raw data and all used scripts must be made publicly available. However, even that does not guarantee reusability due to the variety of issues described in the paper. To combat that, we suggest some processed files should be made available whenever possible.

As you say, this is not always realistic, but we believe that it should be something that we strive for as a community to improve access. For example, researchers with poor computational resources may not be

able to replicate the study to achieve the processed files.

Overall, I think this manuscript does not meet the magazine's requirements for innovation.

We believe the manuscript is innovative because, to our knowledge, this is the first attempt to bring perspectives of plant, animal, and microbial scientists, working on topics related to breeding, bioinformatics, physiology, genetics, etc. together. Such collaboration between agricultural communities in one forum has, to date, not been done to address data reuse.

Further, while reuse has been assessed (as cited in the paper), there have been no focused recommendations for different reuse stakeholders from experts with diverse scientific roles. Our team includes early-career and senior researchers from the public and private sectors. Collectively, we have experience from production to biocuration which allows us unique insight into all aspects of hands-on genomic data reuse. The paper also links to a living document on GitHub to provide data (re)users with recommendations to address the issues raised.

Therefore, in our view, we meet the criteria for GigaScience's review category, namely "systematic and substantial coverage of mature subjects, evaluations of progress in specified areas, and/or critical assessments of emerging technologies".
