# Supplementary material for: Data reuse in agricultural genomics research: challenges and recommendations
Source: Gigascience. 2025 Jan 13;14:giae106. doi: 10.1093/gigascience/giae106 (PMC11727710; doi:10.1093/gigascience/giae106)
Supplement: giae106_Response_to_Reviewer_Comments_Revision_1 [file giae106_response_to_reviewer_comments_revision_1.pdf]

Author's Response To Reviewer Comments

Dear Editor and Reviewer,
Thank you for your comments and suggested revisions. Your comments have helped to improve the quality of the manuscript. Below, please find point by point comments in response to the revision suggestions.

Best regards,
James Koltes

For the Authors

Response to reviewers 2:

Reviewer #2: I carefully read the revised version of the paper and found that the author made some modifications to the previous version. The author discussed in detail some of the issues currently existing in the reuse of omics data and also provided some suggestions. Undoubtedly, these suggestions are very good and require data submitters and the administrators of data storage websites to better plan and design the storage format of these data, which can indeed promote the reuse of these data by subsequent researchers.

In addition, data submitters should provide a clearer description of the experimental design details involved in the data collection process when submitting data.

We provide comments on this topic at Lines 223 to Line 233-238. We have added a clarifying comment in line 234 to point out that sharing experimental designs and protocols together with produced datasets is greatly beneficial to reuse.

It would also be best to upload some data processing scripts to certain websites to facilitate everyone's understanding of the data processing.

We have addressed this in detail in lines 472-483. We have now added a statement to make our suggestion of uploading complete scripts clearer (lines 474 – 476):

"Therefore, it is vital that detailed computational experimental methods used in the study (i.e., scripts) are made available alongside the publication, for example deposited on GitHub."

For the data uploaded by the authors, many database administrators or reviewers find it difficult to verify, as downloading such a large amount of data not only takes time but also consumes considerable computational resources. Reviewers may assess the reliability of the results based on some analysis scripts related to the data.

We address this at lines 422-433. Particularly, we do propose the potential solution of automated checks of data for reviewers/editors.

Additionally, another significant issue in data utilization is that some researchers are reluctant to share their valuable data.

We briefly discuss the evolution of researchers' reuse behaviour in the Introduction (lines 17-22). We deem this issue to be sufficiently addressed in detail by Sielemann et al. (2020) when it comes to an individual's decision to share. We do not have additional suggestions for any agricultural research stakeholders to address the problem as the current sharing requirements and benefits are sufficient to convince most researchers to share their data (lines 20-22). We have added a clarifying statement in line 23.
We also address in detail the issue of sharing data with an industry stakeholder (lines 539-548).

Furthermore, addressing issues such as how to eliminate batch effects in different data processing processes is also an important consideration for data reuse.

This topic is addressed directly in lines 244-246.

The author could provide readers with some suggestions on these aspects.

We have added examples of helpful tools for the handling of batch effects to our address of the topic in lines 244-246:

1. Leek JT, Johnson WE, Parker HS, Jaffe AE, Storey JD. The sva package for removing batch effects and other unwanted variation in high-throughput experiments. Bioinformatics. 2012; doi: 10.1093/bioinformatics/bts034.

2. Parts L, Stegle O, Winn J, Durbin R. Joint Genetic Analysis of Gene Expression Data with Inferred Cellular Phenotypes. Storey JD, editor. PLoS Genet. 2011; doi: 10.1371/journal.pgen.1001276.

3. Stegle O, Parts L, Durbin R, Winn J. A Bayesian Framework to Account for Complex Non-Genetic Factors in Gene Expression Levels Greatly Increases Power in eQTL Studies. Regev A, editor. PLoS Comput Biol. 2010; doi: 10.1371/journal.pcbi.1000770.
