# Supplementary material for: Data reuse in agricultural genomics research: challenges and recommendations
Source: Gigascience. 2025 Jan 13;14:giae106. doi: 10.1093/gigascience/giae106 (PMC11727710; doi:10.1093/gigascience/giae106)
Supplement: giae106_Reviewer_1_Report_Original_Submission — Dijun Chen -- 8/22/2024 [file giae106_reviewer_1_report_original_submission.pdf]

Reviewer Report

Title: Data reuse in agricultural genomics research: challenges and recommendations

Version: Original Submission **Date:** 8/22/2024

Reviewer name: Dijun Chen

Reviewer Comments to Author:

The manuscript titled "Data reuse in agricultural genomics research: challenges and recommendations" provides an in-depth and timely analysis of the key challenges associated with data reuse in agricultural genomics, offering a set of actionable recommendations to improve these practices. The authors successfully identify and discuss critical obstacles such as data standards, metadata deficiencies, data interoperability, and equity issues, contributing valuable insights to the ongoing discourse in this area. While the manuscript is well-structured and presents a thorough examination of the subject, there are several areas that require further attention:Major Points:1. Lines 53-57: The manuscript references several major genomic data centers, but it omits the National Genomics Data Center (NGDC, https://ngdc.cncb.ac.cn/) and the Genome Sequence Archive (GSA, https://ngdc.cncb.ac.cn/gsa/). These should be included to provide a more comprehensive overview of available resources.2. Line 65: The examples of data reuse provided in the manuscript are helpful, but additional references would further strengthen this section. Consider including more examples from plant genomics, such as those found in these studies: 10.1093/nar/gkad706, 10.1093/nar/gkac930, and 10.1038/s41467-022-30770-1. The authors could explore more ...3. While the manuscript covers a broad spectrum of challenges, certain sections would benefit from more in-depth analysis. The discussion on equity issues, for instance, is somewhat superficial. Expanding this section with detailed examples or case studies would provide a more robust understanding of the equity-related challenges in data reuse.4. The manuscript would be significantly strengthened by incorporating specific case studies or examples where data reuse has been successfully implemented in agricultural genomics. These practical illustrations would enhance the manuscript's applicability and offer readers tangible insights into the implementation of the recommendations.5. The introduction effectively sets the stage by underscoring the importance of data reuse in the context of open science. However, it would benefit from a brief historical overview of the evolution of data reuse practices, especially within the agricultural genomics domain. This context would help readers appreciate the current state of data reuse and the progress made thus far.6. The discussion on metadata deficiencies is well-crafted, but it could be further enriched by addressing recent advancements in metadata standards. Discussing how these innovations could mitigate current challenges would provide a more balanced and forward-looking perspective.7. The recommendations offered are thorough and relevant, yet the manuscript would be more practical if it distinguished between short-term and long-term solutions. This distinction would help readers and stakeholders prioritize actions based on their available resources and infrastructure.

Level of Interest

Please indicate how interesting you found the manuscript: Choose an item.

Quality of Written English

Please indicate the quality of language in the manuscript: Choose an item.

Declaration of Competing Interests

Please complete a declaration of competing interests, considering the following questions:

- Have you in the past five years received reimbursements, fees, funding, or salary from an organisation that may in any way gain or lose financially from the publication of this manuscript, either now or in the future?
- Do you hold any stocks or shares in an organisation that may in any way gain or lose financially from the publication of this manuscript, either now or in the future?
- Do you hold or are you currently applying for any patents relating to the content of the manuscript?
- Have you received reimbursements, fees, funding, or salary from an organization that holds or has applied for patents relating to the content of the manuscript?
- Do you have any other financial competing interests?
- Do you have any non-financial competing interests in relation to this paper?

If you can answer no to all of the above, write 'I declare that I have no competing interests' below. If your reply is yes to any, please give details below.

I declare that I have no competing interests

I agree to the open peer review policy of the journal. I understand that my name will be included on my report to the authors and, if the manuscript is accepted for publication, my named report including any attachments I upload will be posted on the website along with the authors' responses. I agree for my report to be made available under an Open Access Creative Commons CC-BY license (http://creativecommons.org/licenses/by/4.0/). I understand that any comments which I do not wish to be included in my named report can be included as confidential comments to the editors, which will not be published.

Choose an item.

To further support our reviewers, we have joined with Publons, where you can gain additional credit to further highlight your hard work (see: https://publons.com/journal/530/gigascience). On publication of this paper, your review will be automatically added to Publons, you can then choose whether or not to claim your Publons credit. I understand this statement.

Yes Choose an item.
