# Supplementary material for: Data reuse in agricultural genomics research: challenges and recommendations
Source: Gigascience. 2025 Jan 13;14:giae106. doi: 10.1093/gigascience/giae106 (PMC11727710; doi:10.1093/gigascience/giae106)
Supplement: giae106_Reviewer_2_Report_Original_Submission — Xuanmin Guang, Ph.D. -- 8/25/2024 [file giae106_reviewer_2_report_original_submission.pdf]

Reviewer Report

Title: Data reuse in agricultural genomics research: challenges and recommendations

Version: Original Submission **Date:** 8/25/2024

Reviewer name: Xuanmin Guang, Ph.D.

Reviewer Comments to Author:

In this manuscript, the authors explored the challenges and recommendations regarding data reuse. It identifies limitations related to data standards, metadata deficiencies, data interoperability, data ownership, data availability, user skill levels, resource availability, and equity issues, and suggests solutions that relevant stakeholders can implement. Additionally, the article offers an optimistic outlook on the future of data reuse in genomics and transcriptomics. Although the authors had concluded the current problems during the data usage, but the most important things were solved to these problems, they didn't give any solutions. Currently, we have different type of data, eg. RNA-data, Protein data, Metabolic data, Stero-seq data, with different type of data, we need individual solutions for each type. In the part of data formatting, the authors didn't clarify the other types of data not only for the normal genome data. I think they should add this part. In the manuscript, the author said that the process files need also should be upload, I think this can't be realistic. Researchers can upload the data, as well as with some script, otherwise the database need a large storage. Overall, I think this manuscript does not meet the magazine's requirements for innovation.

Level of Interest

Please indicate how interesting you found the manuscript: Choose an item.

Quality of Written English

Please indicate the quality of language in the manuscript: Choose an item.

Declaration of Competing Interests

Please complete a declaration of competing interests, considering the following questions:

- Have you in the past five years received reimbursements, fees, funding, or salary from an organisation that may in any way gain or lose financially from the publication of this manuscript, either now or in the future?
- Do you hold any stocks or shares in an organisation that may in any way gain or lose financially from the publication of this manuscript, either now or in the future?
- Do you hold or are you currently applying for any patents relating to the content of the manuscript?

- Have you received reimbursements, fees, funding, or salary from an organization that holds or has applied for patents relating to the content of the manuscript?
- Do you have any other financial competing interests?
- Do you have any non-financial competing interests in relation to this paper?

If you can answer no to all of the above, write 'I declare that I have no competing interests' below. If your reply is yes to any, please give details below.

I declare that I have no competing interests

I agree to the open peer review policy of the journal. I understand that my name will be included on my report to the authors and, if the manuscript is accepted for publication, my named report including any attachments I upload will be posted on the website along with the authors' responses. I agree for my report to be made available under an Open Access Creative Commons CC-BY license (http://creativecommons.org/licenses/by/4.0/). I understand that any comments which I do not wish to be included in my named report can be included as confidential comments to the editors, which will not be published.

Choose an item.

To further support our reviewers, we have joined with Publons, where you can gain additional credit to further highlight your hard work (see: https://publons.com/journal/530/gigascience). On publication of this paper, your review will be automatically added to Publons, you can then choose whether or not to claim your Publons credit. I understand this statement.

Yes Choose an item.
