# Supplementary material for: Data reuse in agricultural genomics research: challenges and recommendations
Source: Gigascience. 2025 Jan 13;14:giae106. doi: 10.1093/gigascience/giae106 (PMC11727710; doi:10.1093/gigascience/giae106)
Supplement: giae106_Reviewer_2_Report_Revision_1 — Xuanmin Guang, Ph.D. -- 10/8/2024 [file giae106_reviewer_2_report_revision_1.pdf]

Reviewer Report

Title: Data reuse in agricultural genomics research: challenges and recommendations

Version: Revision 1 **Date:** 10/8/2024

Reviewer name: Xuanmin Guang, Ph.D.

Reviewer Comments to Author:

I carefully read the revised version of the paper and found that the author made some modifications to the previous version. The author discussed in detail some of the issues currently existing in the reuse of omics data and also provided some suggestions. Undoubtedly, these suggestions are very good and require data submitters and the administrators of data storage websites to better plan and design the storage format of these data, which can indeed promote the reuse of these data by subsequent researchers. In addition, data submitters should provide a clearer description of the experimental design details involved in the data collection process when submitting data. It would also be best to upload some data processing scripts to certain websites to facilitate everyone's understanding of the data processing.For the data uploaded by the authors, many database administrators or reviewers find it difficult to verify, as downloading such a large amount of data not only takes time but also consumes considerable computational resources. Reviewers may assess the reliability of the results based on some analysis scripts related to the data. Additionally, another significant issue in data utilization is that some researchers are reluctant to share their valuable data.Furthermore, addressing issues such as how to eliminate batch effects in different data processing processes is also an important consideration for data reuse. The author could provide readers with some suggestions on these aspects.

Level of Interest

Please indicate how interesting you found the manuscript: Choose an item.

Quality of Written English

Please indicate the quality of language in the manuscript: Choose an item.

Declaration of Competing Interests

Please complete a declaration of competing interests, considering the following questions:

- Have you in the past five years received reimbursements, fees, funding, or salary from an organisation that may in any way gain or lose financially from the publication of this manuscript, either now or in the future?
- Do you hold any stocks or shares in an organisation that may in any way gain or lose financially from the publication of this manuscript, either now or in the future?

- Do you hold or are you currently applying for any patents relating to the content of the manuscript?
- Have you received reimbursements, fees, funding, or salary from an organization that holds or has applied for patents relating to the content of the manuscript?
- Do you have any other financial competing interests?
- Do you have any non-financial competing interests in relation to this paper?

If you can answer no to all of the above, write 'I declare that I have no competing interests' below. If your reply is yes to any, please give details below.

I declare that I have no competing interests

I agree to the open peer review policy of the journal. I understand that my name will be included on my report to the authors and, if the manuscript is accepted for publication, my named report including any attachments I upload will be posted on the website along with the authors' responses. I agree for my report to be made available under an Open Access Creative Commons CC-BY license (http://creativecommons.org/licenses/by/4.0/). I understand that any comments which I do not wish to be included in my named report can be included as confidential comments to the editors, which will not be published.

Choose an item.

To further support our reviewers, we have joined with Publons, where you can gain additional credit to further highlight your hard work (see: https://publons.com/journal/530/gigascience). On publication of this paper, your review will be automatically added to Publons, you can then choose whether or not to claim your Publons credit. I understand this statement.

Yes Choose an item.
